# Luminescent Behavior of Zn(II) and Mn(II) Halide Derivatives of 4-Phenyldinaphtho[2,1-d:1′,2′-f][1,3,2]dioxaphosphepine 4-Oxide and Single-Crystal X-ray Structure Determination of the Ligand

**DOI:** 10.3390/molecules29010239

**Published:** 2024-01-01

**Authors:** Valentina Ferraro, Jesús Castro, Marco Bortoluzzi

**Affiliations:** 1 Dipartimento di Scienze Molecolari e Nanosistemi, Università Ca’ Foscari Venezia, 30172 Mestre, VE, Italy; valentina.ferraro@unive.it; 2 Departamento de Química Inorgánica, Facultade de Química, Edificio de Ciencias Experimentais, Universidade de Vigo, 36310 Vigo, Galicia, Spain; jesusc@uvigo.gal

**Keywords:** phosphonates, BINOL, zinc(II), manganese(II), luminescence, dual emission

## Abstract

The two enantiomers of chiral phosphonate 4-phenyldinaphtho[2,1-d:1′,2′-f][1,3,2]dioxaphosphepine 4-oxide, O=PPh(BINOL), were synthesized from the proper 1,1′-bi-2-naphtol (BINOL) enantiomer and characterized. The structure of the (*S*)-enantiomer was elucidated by means of single-crystal X-ray diffraction. The reaction with anhydrous ZnBr_2_ afforded complexes having the general formula [ZnBr_2_{O=PPh(BINOL)}_2_] that showed intense fluorescence centered in the near-UV region rationalized on the basis of TD-DFT calculations. The corresponding Mn(II) complexes with the general formula [MnX_2_{O=PPh(BINOL)}_2_] (X = Cl, Br) exhibited dual emission upon excitation with UV light, with the relative intensity of the bands dependent upon the choice of the halide. The highest energy transition is comparable with that of the Zn(II) complex, while the lowest energy emission falls in the red region of the spectrum and is characterized by lifetimes in the hundreds of microseconds range. Although the emission at lower energy can also be achieved by direct excitation of the metal center, the luminescence decay curves suggest that the band in the red range is possibly derived from BINOL-centered excited states populated by intersystem crossing.

## 1. Introduction

The enantiomers of 1,1′-bi-2-naphtol (BINOL) and related species play a key role in asymmetric organic synthesis and catalysis [1,2,3,4,5,6,7,8,9,10,11,12,13]. A recent noticeable example is constituted by the integration of chiral BINOL-phosphoric acid and Cu(II)-porphyrin modules into a single framework, able to promote the asymmetric α-benzylation of aldehydes via visible-light-induced photothermal conversion [14]. Actual advanced applications of BINOL-based compounds include supramolecular enantiorecognition, which is also based on luminescence measurements, and the development of materials with chiral optical properties [15,16,17,18,19,20,21]. Recent examples in this field are chiroptical switches constituted by tetrahedral boron BINOL derivatives containing fluorene- or dibenzosuberenone-substituted pyridinylhydrazone moieties that exhibit noticeable chiroptical changes after visible light irradiation [22].

Even though several BINOL derivatives of synthetic and technological interest contain trivalent or pentavalent phosphorus, such as phosphonites and phosphoric acid derivatives, the chiral phosphonate 4-phenyldinaphtho[2,1-d:1′,2′-f][1,3,2]dioxaphosphepine 4-oxide, O=PPh(BINOL), was subjected to quite limited investigation. Its synthesis, first reported in 1990, was based on the reaction between BINOL and phenylphosphonic dichloride in benzene in the presence of triethylamine as a base. The compound and related species were investigated as possible new organophosphorus pesticides and insecticides [23]. According to a 2023 Chinese patent [24], the same product can be obtained from BINOL and diethyl phenylphosphonate in the presence of trifluoromethylsulfonic anhydride, dimethyl sulfoxide, and tetrabutylammonium bromide. The synthesis of *P*-stereogenic 2′-hydroxy-[1,1′-binaphthalen]-2-yl alkyl(phenyl)phosphinates from O=PPh(BINOL) was achieved by reacting this species with Grignard reagents, methylmagnesium bromide, and isopropylmagnesium bromide, in particular [25].

Compounds containing the [O=P]-donor moiety proved to be a viable choice to isolate luminescent Mn(II) complexes, sometimes behaving as multifunctional materials [26,27,28,29,30,31]. Another interesting metal center to be combined with [O=P]-donor ligands for optics is zinc. For instance, non-linear optical properties were observed for [ZnCl_2_(O=PPh_3_)_2_] [32], and Zn(II) halide complexes with *N*,*N*,*N*′,*N*′-tetramethyl-*P*-indol-1-ylphosphonic diamide showed intense green phosphorescence [33]. Given our interest in the preparation of luminescent metal complexes with phosphonates and cyclic organophosphorus compounds [34,35,36,37], we synthesized the two enantiomers of O=PPh(BINOL), and we started investigating the luminescent properties of the related Zn(II) and Mn(II) halide complexes. The preliminary results, together with the single-crystal X-ray structure determination of (*S*)-O=PPh(BINOL), are provided in this communication.

## 2. Results and Discussion

**Scheme 1:**
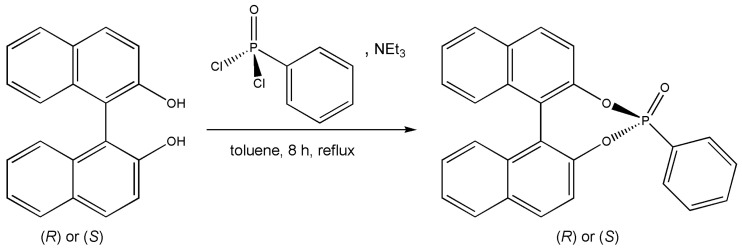
Synthesis of (*R*)- and (*S*)-O=PPh(BINOL).

The two O=PPh(BINOL) enantiomers were isolated in good yield and purity by reacting the BINOL enantiomers with O=PPhCl_2_ in the presence of triethylamine as a proton scavenger (Scheme 1). The formation of the desired products was confirmed by elemental analysis data and NMR spectroscopy. The ^1^H NMR spectra, comprised between 8.2 and 7.0 ppm, are coherent with the presence of 17 aromatic protons. Despite the superposition of some resonances, most of the doublets related to the hydrogen atoms in the 3, 3′, 4, 4′, 5, 5′, and 8, 8′ positions of the BINOL fragment can be distinguished. The ^13^C{^1^H} NMR spectra confirm the lack of equivalence of the two naphthyl rings in O=PPh(BINOL), which show 15 CH and 9 C_ipso_ resonances, with some coupled with the ^31^P nucleus. The ^31^P{^1^H} NMR spectra are composed of a single sharp signal around 27 ppm. Selected NMR spectra are provided as Supplementary Materials Figures S1–S4. The ν_P=O_ stretching is tentatively assigned to the strong band at 1280 cm^−1^ also on the basis of the comparison with the related Zn(II) complex described below.

The values of specific rotation [α]^20^_D_ measured in acetone for the two enantiomers are −326 for (*R*)-O=PPh(BINOL) and +326 for (*S*)-O=PPh(BINOL), which are much higher than those measured for the enantiomers of free BINOL under the same experimental conditions (±30), an effect attributable to the more rigid structure generated by the formation of the {C_4_O_2_P} ring. It is worth noting that the specific rotation is inverted, moving from a BINOL enantiomer to the related O=PPh(BINOL) enantiomer.

**Figure 1:**
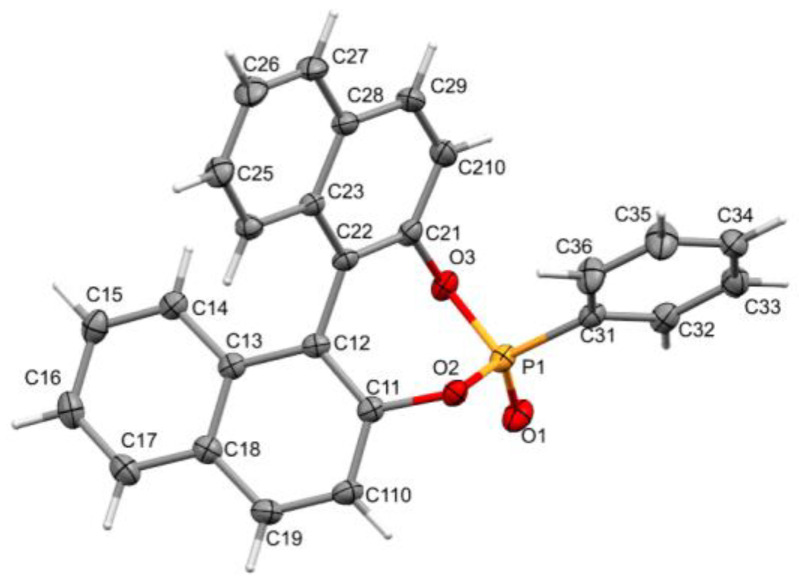
Single-crystal X-ray structure of (*S*)-O=PPh(BINOL). Selected bond lengths [Å]: P(1)-O(1) 1.4619(13), P(1)-O(2) 1.6028(12), P(1)-O(3) 1.5985(12), P(1)-C(31) 1.7770(17), O(2)-C(11) 1.407(2), O(3)-C(21) 1.4047(19), C(11)-C(12) 1.378(2), C(21)-C(22) 1.376(2), C(12)-C(22) 1.486(2). Selected angles [°]: O(1)-P(1)-O(2) 117.13(8), O(1)-P(1)-O(3) 109.81(7), O(3)-P(1)-O(2) 102.79(6), O(1)-P(1)-C(31) 114.91(8), O(3)-P(1)-C(31) 108.85(7), O(2)-P(1)-C(31) 102.37(7), C(11)-O(2)-P(1) 118.18(10), C(21)-O(3)-P(1) 119.07(10).

Crystals of (*S*)-O=PPh(BINOL) suitable for single-crystal X-ray diffraction were collected from dichloromethane/diethyl ether solutions. A picture of the structure is provided in Figure 1. Crystal data and structure refinement are summarized in the Supplementary Materials Table S1, while selected bond lengths and angles are reported in the caption of Figure 1. The chiral BINOL derivative crystallizes in *P*2_1_2_1_2_1_, one of the so-called Söhncke space groups. The Flack parameter was refined to a value of −0.004(14) confirming the absolute structure [38]. The environment of the phosphorus atom is tetrahedral, with angles ranging from 102.37(7) to 117.13(8)°; the former corresponds to the O-P-O angle in the seven-membered ring of the dioxaphosphepine. The P-O and P-C distances are in accordance with the nature of the bonds, with the double P=O bond being about 0.14 Å shorter than the single P-O bonds. The P-C bond length, 1.777(2) Å, is, as expected, longer than that found in the P(III) compound 8-phenyldinaphtho[2,1-d:1′,2′-f][1,3,2]dioxaphosphepine [39].

**Figure 2:**
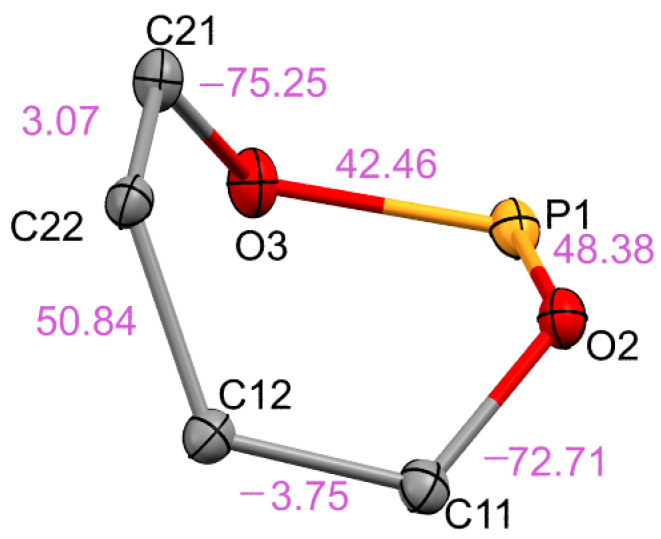
{PO_2_C_4_} 7-membered ring of (S)-O=PPh(BINOL) with torsion angles (°).

The two naphthalene rings (10 membered) form a dihedral angle of 59.88(5)°. The 7-membered ring is, as expected, highly puckered, with a boat conformation with torsion angles as denoted in Figure 2 [40]. The root mean square deviation for this ring from the best plane is 0.3757 Å. The oxygen atoms are the most deviated, with one of them 0.557(1) Å over and the other 0.532(1) Å below the best plane. This plane and the benzene ring form a dihedral angle of 76.25(6)°, which is different from the quasi-perpendicular disposition, which is equal to 90.4(3)° and found in the P(III) dioxaphosphepine compound [39].

O=PPh(BINOL) behaves as a ligand towards Zn(II), and the reaction between O=PPh(BINOL) and anhydrous ZnBr_2_ allows the isolation of complexes having the general formula [ZnBr_2_{O=PPh(BINOL)}_2_] in yields above 70%. Unfortunately, all the attempts to grow single crystals suitable for X-ray diffraction failed. The proposed formula is, however, supported by elemental analysis data and NMR spectroscopy. The ^1^H NMR spectra, as expected, independent upon the choice of the O=PPh(BINOL) enantiomer, show only resonances in the 8.5–6.5 ppm range related to the phenyl and BINOL moieties, which are shifted and broadened compared to the free ligands. On the other hand, a single resonance around 28 ppm is observable in the ^31^P{^1^H} NMR spectra, which is broadened in comparison to O=PPh(BINOL). Selected spectra are reported in Figures S5 and S6. As a further confirmation, the IR spectra closely resemble those of the free ligands, as observable in Figure S7. The only noticeable change affects the band centered at 1280 cm^−1^ in the free ligands, which was, for this reason, assigned to the ν_P=O_ stretching [41]. The complex is thermally stable up to about 150 °C, then decomposition with mass loss occurs. The related TGA curve is reported in Figure S8.

**Figure 3:**
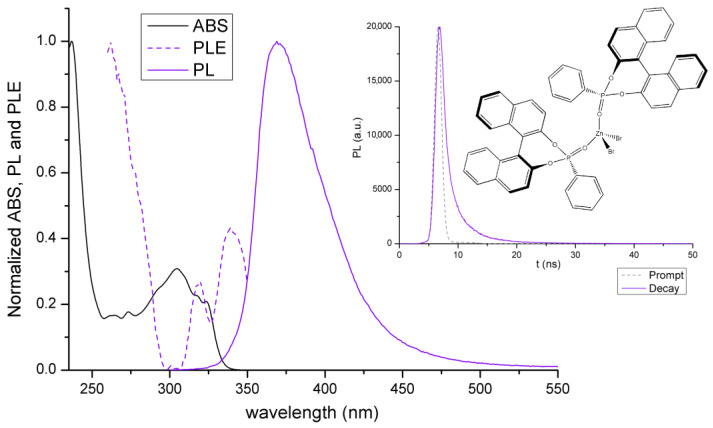
Normalized absorption (ABS, CH_2_Cl_2_ solution, r.t.), emission (PL, solid, r.t., λ_excitation_ = 260 nm), and excitation (PLE, solid, r.t., λ_emission_ = 410 nm) spectra of (*S*,*S*)-[ZnBr_2_{O=PPh(BINOL)}_2_] at room temperature. Luminescence decay curve (solid, r.t., λ_excitation_ = 373 nm, λ_emission_ = 410 nm) of (*S*,*S*)-[ZnBr_2_{O=PPh(BINOL)}_2_].

**Figure 4:**
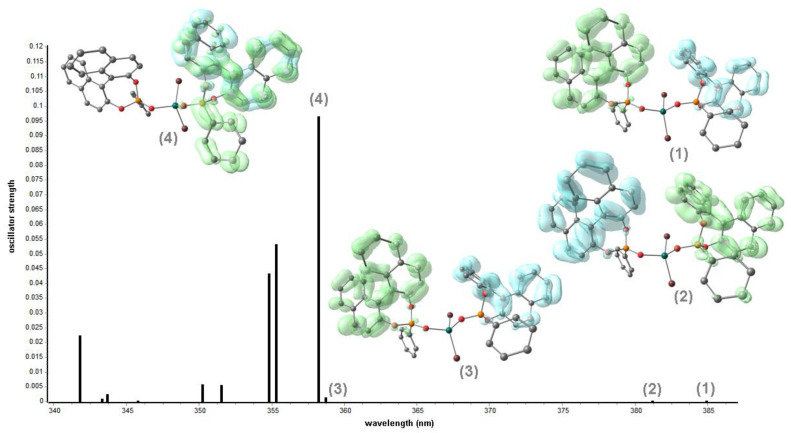
TD-DFT simulated spectrum (C-PCM/r^2^SCAN-3c) of [ZnBr_2_{O=PPh(BINOL)}_2_] with hole (light blue) and electron (green) distributions for selected transitions. Color map: Zn, green; Br, dark red; O, red; P, orange; C, grey. Hydrogen atoms are omitted for clarity. Surface isovalue = 0.001 a.u.

Dichloromethane solutions of the two enantiomers of [ZnBr_2_{O=PPh(BINOL)}_2_] absorb radiation for wavelengths below 340 nm, as observable, for instance, in Figure 3. SOC-corrected TD-DFT calculations at the C-PCM/r^2^SCAN-3c level (dichloromethane as a continuous medium) predict the most intense absorption at 358 nm, with an underestimation of the energy of around 5%. According to the hole-electron distribution [42], the absorption appears to be essentially related to the presence of the BINOL fragment, and the same consideration is also valid for further bands predicted at longer wavelengths, between 359 and 385 nm, with much lower intensities. In all cases, the participation of triplet configurations is negligible. As observable in Figure 4, the character of the transition changes from ligand-centered for the main absorption to interligand charge transfer for the bands predicted at lower energy.

The [α]^20^_D_ values measured in acetone are ±252, positive for (*S*,*S*)-[ZnBr_2_{O=PPh(BINOL)}_2_] and negative for (*R*,*R*)-[ZnBr_2_{O=PPh(BINOL)}_2_]. Such a result indicates the scarce influence of the coordination on the specific rotation since the [α]^20^_D_ values are roughly proportional to the molar quantity of O=PPh(BINOL) in solution.

**Figure 5:**
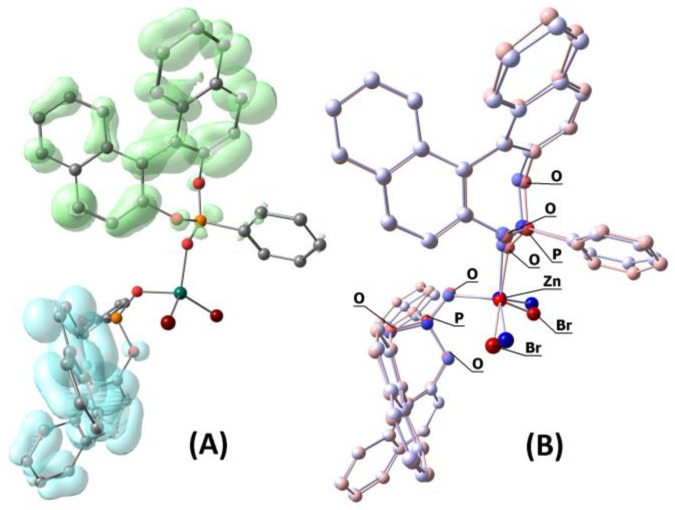
(**A**) TD-DFT optimized (C-PCM/B97-3c) first excited singlet state of [ZnBr_2_{O=PPh(BINOL)}_2_] with hole (light blue) and electron (green) distributions for the S_1_←S_0_ transition (C-PCM-r^2^SCAN-3c). Fluorescence is the reverse process. Color map: Zn, green; Br, dark red; O, red; P, orange; C, grey. Hydrogen atoms are omitted for clarity. Surface isovalue = 0.001 a.u. (**B**) Best superposition of the ground singlet geometry (blue tones) and the first excited singlet geometry (red tones) of [ZnBr_2_{O=PPh(BINOL)}_2_].

The luminescence of dichloromethane solutions of the complexes is hardly appreciable to the human eye. Resolved emission spectra (see, for example, Figure S9) were collected only using quite-opened slits and long acquisition times. On the other hand, the luminescence is easily detectable in the solid state. Solid samples of [ZnBr_2_{O=PPh(BINOL)}_2_] show only an emission band centered at 370 nm after excitation with UV light (Figure 3), coherent with the PL spectra collected in solution and attributed to a fluorescent decay on the basis of the luminescence lifetime, equal to 8 ns. The luminescence quantum yield is 81%. The main luminescence data are summarized in Table 1. Based on the previously described TD-DFT calculations, it can be tentatively supposed that the luminescence exhibited by [ZnBr_2_{O=PPh(BINOL)}_2_] could be ascribed to the population of excited singlet states having an interligand charge transfer nature. Such a hypothesis was confirmed by optimization at the C-PCM/B97-3c level for the lowest energy singlet excited state of [ZnBr_2_{O=PPh(BINOL)}_2_], with subsequent simulation of the transitions at the C-PCM/r^2^SCAN-3c level. The hole-electron distribution, shown in Figure 5, agrees with an interligand charge transfer associated with S_1_→S_0_ radiative decay. The geometry variations between the singlet ground state and the first singlet excited state are quite limited, with the RMSD being 0.223 Å (see also Figure 5).

The synthetic procedure was extended to the preparation of Mn(II) derivatives by replacing ZnBr_2_ with Mn(II) halides. Complexes having the general formula [MnX_2_{O=PPh(BINOL)}_2_] (X = Cl, Br) were isolated with yields higher than 70%. Elemental analyses agree with the proposed general formula. The experimental magnetic moments are in line with the theoretical 5.9 BM expected for high-spin d^5^ derivatives of first-row transition elements. The IR spectra, shown in Figure S10, are strictly similar to those of [ZnBr_2_{O=PPh(BINOL)}_2_], confirming the slight lowering of the ν_P=O_ stretching caused by the coordination. The ^31^P{^1^H} NMR spectra of the [MnX_2_{O=PPh(BINOL)}_2_] complexes are broadened by paramagnetic relaxation, particularly for the bromo-derivative; however, a single resonance centered in the 27–28 ppm range was detected, which is in line with the value reported for [ZnBr_2_{O=PPh(BINOL)}_2_]. The nuclearity of the Mn(II) complexes cannot be unambiguously defined since all the attempts to grow single crystals suitable for X-ray diffraction were unsuccessful. The thermal behavior of [MnBr_2_{O=PPh(BINOL)}_2_] is strictly comparable with that of the analogous Zn(II) bromo-derivative with decomposition around 150 °C. On the other hand, despite the melting point in the same temperature range (160 °C), the chloro-complex [MnCl_2_{O=PPh(BINOL)}_2_] did not exhibit meaningful mass loss for temperatures below 300 °C (Figure S8).

As previously described for the Zn(II) enantiomers, the specific rotation values appear dependent upon the molar quantity of the chiral phosphonate in solution without meaningful influence of the Mn-O bonds. [α]^20^_D_ is ±282 for (*S*,*S*)- and (*R*,*R*)-[MnCl_2_{O=PPh(BINOL)}_2_] and ±248 for (*R*,*R*)- and (*S*,*S*)-[MnBr_2_{O=PPh(BINOL)}_2_].

**Figure 6:**
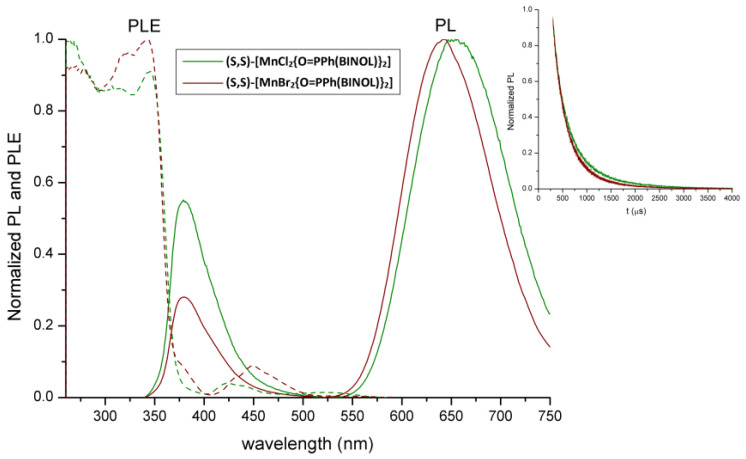
Normalized emission (PL, solid, r.t., λ_excitation_ = 310 nm, solid lines) and excitation (PLE, solid, r.t., λ_emission_ = 650 nm, dotted lines) spectra of (*S*,*S*)-[MnX_2_{O=PPh(BINOL)}_2_] (X = Cl, Br) at room temperature. Luminescence decay curves (solid, r.t., λ_excitation_ = 290 nm, λ_emission_ = 640 nm) of (*S*,*S*)-[MnX_2_{O=PPh(BINOL)}_2_] (X = Cl, Br).

As for the Zn(II) complex, the luminescence measurements were limited to the solid state (Table 1). The PL spectra of [MnX_2_{O=PPh(BINOL)}_2_] (Figure 6) are composed of two different bands: one centered in the UV region and similar to that already described for [ZnBr_2_{O=PPh(BINOL)}_2_] (λ_max_ = 380 nm), while the other centered between 641 and 653 nm, with FWHM values in the 2500–2600 cm^−1^ range. No emission in the green region attributable to the Mn(II) ^4^T_1_(^4^G)→^6^A_1_(^6^S) transition in a tetrahedral environment was detected. The PLE spectra (Figure 6) reveal that the lowest energy emission can be achieved with UV light shorter than 380 nm, but also using wavelengths in the 410–500 nm range. The higher energy bands in the PLE spectra are almost in part associated with the excitation of the coordinated O=PPh(BINOL) ligands, while those at lower energy are in line with direct Mn(II) excitation [43,44].

It is worth noting that the relative intensity of Mn(II) excitation compared to the ligands′ excitation depends upon the choice of the halide due to the increased spin-orbit coupling moving from X = Cl to X = Br that relaxes the selection rules for the metal-centered transitions [45]. The relative intensity of the PL bands is also dependent upon the nature of the halide, since the emission in the red range is relatively more intense for the enantiomers of [MnBr_2_{O=PPh(BINOL)}_2_] (Figure 6). The increased spin-orbit coupling appears to also be involved in this case, favoring the population of lower-energy excited states by intersystem crossing. The relative increase of the emission at a lower energy has, as a consequence, a noticeable reduction of the luminescence quantum yield, which drops from 48% for [MnCl_2_{O=PPh(BINOL)}_2_] to 14% for [MnBr_2_{O=PPh(BINOL)}_2_]. Such an outcome was expected on the basis of the energy gap law [46], but it also suggests that the localization of the lower energy excited state could be in molecular regions where the electronic-vibrational coupling is favorable, such as the coordinated phosphonates.

The band in the red range is not present in the PL spectrum of [ZnBr_2_{O=PPh(BINOL)}_2_]; it is also absent in the PL spectrum of the free ligand collected at room temperature (Figure S11). Emissions in the yellow-red range can be associated with radiative decay from Mn(II) excited states in complexes with a coordination number greater than four, for instance, five-coordinated [47,48,49,50], but also with phosphorescence from ligand-centered excited states populated by intersystem crossing [51,52]. The fact that the red emission could also be achieved by direct Mn(II) excitation does not allow an unambiguous discrimination since the excited states of the metal center could populate low-lying ligand-centered excited states. TD-DFT calculations on [ZnBr_2_{O=PPh(BINOL)}_2_] in the triplet state configuration indicate the possibility of a triplet→singlet BINOL-centered emission in the red range with λ_emission_ calculated at 607 nm. The hole-electron distribution related to the transition is shown in Figure S12. The energy gap between the optimized geometries of [ZnBr_2_{O=PPh(BINOL)}_2_] in triplet and singlet states is identical to the one obtained between the geometries of [MnBr_2_{O=PPh(BINOL)}_2_] in octet and sextet states (55.7 kcal mol^−1^); thus, the same transition appears possible also for the Mn(II) complexes. The role of the BINOL fragments in the octet configuration is highlighted by the spin density plots shown in Figure S12. It is, however, worth noting that DFT calculations on the tetrahedral mononuclear [MnBr_2_{O=PPh(BINOL)}_2_] and on the dimer [MnBr(μ-Br){O=PPh(BINOL)}_2_]_2_, where the metal center is five-coordinated, indicate that the formation of the latter species is possible. The energy variation (electronic energy + nuclear repulsion) for the dimerization is negative by about −25.7 kcal mol^−1^. Despite the reduction in molecularity, the estimated Gibbs energy variation is slightly negative, being about −2.3 kcal mol^−1^ (Figure S13).

The localization of the emitting states can be tentatively supposed from the luminescence decay curves of [MnCl_2_{O=PPh(BINOL)}_2_] and [MnBr_2_{O=PPh(BINOL)}_2_]. As observable in Figure 6, the decays are quite similar, with average lifetimes (τ_av_) of 422 μs for [MnCl_2_{O=PPh(BINOL)}_2_] and 335 μs for [MnBr_2_{O=PPh(BINOL)}_2_]. The bi-exponential fit of the curves reveals the presence of a common component (τ_1_), equal to 220 μs (58%) for X = Cl and to 216 μs (62%) for X = Br. The second component (τ_2_) is equal to 700 μs (42%) for X = Cl and 530 μs (38%) for X = Br. The presence of bi-exponential decay could be associated with the superimposition of different emission bands, but such a hypothesis appears unlikely considering the shape and FWHM values of the emissions in the red range. The bi-exponential decays are thus tentatively ascribed to solid-state effects in the samples. In every case, the scarce influence of the nature of the halide on the lifetime is more in line with a ligand-centered process involving the BINOL fragments than a metal-centered one. It appears, therefore, that the main role of Mn(II) is to favor intersystem crossing among phosphonate-centered excited states, probably due to the intermediate population of Mn(II) excited levels. The influence of the coordinated halides on the luminescence quantum yield can be thus interpreted considering that the acceleration of the intersystem crossing given by the presence of the bromo-ligands favors the population of the lower energy long-lived excited state. The low quantum yield is caused by the efficient vibrational decay in this state.

## 3. Experimental Section

### 3.1. Materials and Methods

Commercial solvents (Merck, Darmstadt, Germany) were purified, as described in the literature [53]. Anhydrous MX_2_ halides (M = Mn, X = Cl, Br; M = Zn, X = Br) were purchased from Alfa Aesar (Ward Hill, MA, USA) and Merck. The enantiomers of BINOL and the other organic reactants were Merck products. All the syntheses were carried out under an inert atmosphere, working in a MBraun Labstar glove-box with a MB 10 G gas purifier (Garching, Germany) filled with N_2_ and equipped for organic and inorganic syntheses.

Elemental analyses (C, H) were carried out using an Elementar Unicube microanalyzer (Langenselbold, Germany). The halide content was determined using Mohr’s method [54].

Magnetic susceptibilities were measured on solid samples at 298 K using an MK1 magnetic susceptibility balance (Sherwood Scientific Ltd., Cambridge, UK, magnetic field strength 3.5 kGauss) and corrected for diamagnetic contribution using tabulated Pascal’s constants [55].

Melting points were registered using a FALC 360 D instrument equipped with a camera. Thermogravimetric analyses (TGA) were carried out under am N_2_ atmosphere with a Perkin-Elmer TGA 4000 instrument (Waltham, MA, USA). The heating rate was set at 20 °C min^−1^ from 40 °C up to 500 °C.

IR spectra were collected in the 4000–400 cm^−1^ range using a Perkin-Elmer Spectrum One spectrophotometer. Mono- and bidimensional nuclear magnetic resonance (NMR) spectra were collected employing a Bruker Avance 400 instrument (Billerica, MA, USA) operating at 400.13 MHz of ^1^H resonance. ^1^H NMR spectra are referred to as the partially non-deuterated fraction of the solvent, itself referred to as tetramethylsilane. ^31^P{^1^H} NMR resonances refer to 85% H_3_PO_4_ in water. ^13^C{^1^H} NMR spectra are quoted with respect to the signal of the solvent, itself referred to as tetramethylsilane.

### 3.2. Synthesis of 4-Phenyldinaphtho[2,1-d:1′,2′-f][1,3,2]dioxaphosphepine 4-Oxide, O=PPh(BINOL)

The two enantiomers of O=PPh(BINOL) were obtained following a modified literature procedure [23]. In a typical preparation, a solution containing 5.0 mmol (1.432 g) of (*R*)- or (*S*)-BINOL and 0.7 mL of phenylphosphonic dichloride (0.975 g, 5.0 mmol) in dry toluene (30 mL) was prepared under an inert atmosphere, and then 1.4 mL of triethylamine (10.0 mmol) were slowly added. The mixture was then refluxed for 8 h and subsequently stirred overnight. The by-product triethylammonium chloride was separated by centrifugation, then the solution was filtered on cotton, and the solvent was evaporated under reduced pressure. The addition of diethyl ether (10 mL) caused the separation of a white solid that was filtered, washed with 5 mL of diethyl ether, and dried under vacuum. Yields: (*R*)-O=PPh(BINOL), 80% (1.633 g); (*S*)-O=PPh(BINOL), 77% (1.577 g).

*Characterization of O=PPh(BINOL)*. Anal. Calcd for C_26_H_17_O_3_P (408.39 g mol^−1^, %): C, 76.47; H, 4.20. Found for (*R*)-O=PPh(BINOL) (%): C, 76.16; H, 4.22. Found for (*S*)-O=PPh(BINOL) (%): C, 76.28; H, 4.22. ^1^H NMR (CDCl_3_, 300 K, both enantiomers): δ 8.10 (d, 1H, *J*_HH_ = 8.8 Hz, BINOL), 8.01 (d, 1H, *J*_HH_ = 8.2 Hz, BINOL), 7.96 (d, 1H, *J*_HH_ = 8.3 Hz, BINOL), 7.87 (d, 1H, *J*_HH_ = 8.8 Hz, BINOL), 7.69 (d, 1H, *J*_HH_ = 8.8 Hz, BINOL), 7.68–7.57 (m, 3H, BINOL+Ph), 7.56–7.50 (m, 2H, BINOL+Ph), 7.49 (d, 1H, *J*_HH_ = 9.0 Hz, BINOL), 7.44–7.30 (m, 5H, BINOL+Ph), 7.04 (dd, 1H, *J*_HH_ = 8.8 Hz, *J*_HH_ = 0.7 Hz, BINOL). ^13^C{^1^H} NMR (CDCl_3_, 300 K, both enantiomers): δ 147.60 (d, *J*_CP_ = 10.2 Hz, C_ipso_), 145.83 (d, *J*_CP_ = 9.6 Hz, C_ipso_), 133.56 (d, *J*_CP_ = 3.0 Hz, CH), 132.52 (s, CH), 132.49 (d, *J*_CP_ = 1.4 Hz, C_ipso_), 132.45 (d, *J*_CP_ = 1.6 Hz, C_ipso_), 132.42 (s, CH), 131.94 (d, *J*_CP_ = 1.1 Hz, C_ipso_), 131.56 (d, *J*_CP_ = 1.1 Hz, C_ipso_), 131.37 (d, *J*_CP_ = 1.1 Hz, CH), 130.72 (s, CH), 128.58 (s, CH), 128.52 (s, CH), 128.48 (s, CH), 128.37 (s, CH), 127.32 (s, CH), 127.01 (s, CH), 126.73 (s, CH), 125.76 (s, CH), 124.85 (d, *J*_CP_ = 186.9 Hz, C_ipso_), 121.89 (d, *J*_CP_ = 2.6 Hz, C_ipso_), 121.75 (d, *J*_CP_ = 2.0 Hz, C_ipso_), 121.28 (d, *J*_CP_ = 2.2 Hz, CH), 120.89 (d, *J*_CP_ = 3.3 Hz, CH). ^31^P{^1^H} NMR (CDCl_3_, 300 K, both enantiomers) δ 27.1 (FWHM = 2 Hz). IR (cm^−1^, both enantiomers): 3110–3020 m/w (aromatic ν_C-H_), 1620–1510 m (aromatic ν_C-C_), 1325 m (ν_C-O_), 1280 (ν_P=O_).

### 3.3. Synthesis of [MX_2_{O=PPh(BINOL)}_2_] (M = Zn, X = Br; M = Mn, X = Cl, Br)

In a typical preparation, 2.0 mmol (0.817 g) of (*R*)- or (*S*)-O=PPh(BINOL) were reacted overnight under an inert atmosphere with 1.0 mmol of the proper anhydrous MX_2_ precursor in freshly distilled ethanol (20 mL) at room temperature. After the removal of the solvent under reduced pressure, the residue was dissolved in the minimum amount of dichloromethane, and the solution was cleared by centrifugation. After evaporation, diethyl ether (10 mL) was added, and the products were collected as white powders after filtration, washed with 5 mL of diethyl ether, and dried under vacuum. Yields: (*R*,*R*)-[ZnBr_2_{O=PPh(BINOL)}_2_], 71% (0.740 g); (*S*,*S*)-[ZnBr_2_{O=PPh(BINOL)}_2_], 73% (0.761 g); (*R*,*R*)-[MnCl_2_{O=PPh(BINOL)}_2_], 76% (0.716 g); (*S*,*S*)-[MnCl_2_{O=PPh(BINOL)}_2_], 74% (0.697 g); (*R*,*R*)-[MnBr_2_{O=PPh(BINOL)}_2_], 80% (0.825 g); (*S*,*S*)-[MnBr_2_{O=PPh(BINOL)}_2_], 77% (0.794 g).

*Characterization of [ZnBr_2_{O=PPh(BINOL)}_2_].* Anal. Calcd for C_52_H_34_Br_2_O_6_P_2_Zn (1041.97 g mol^−1^, %): C, 59.94; H, 3.29; Br, 15.34. Found for (*R*,*R*)-[ZnBr_2_{O=PPh(BINOL)}_2_] (%): C, 59.70; H, 3.31; Br, 15.28. Found for (*S*,*S*)-[ZnBr_2_{O=PPh(BINOL)}_2_] (%): C, 59.75; H, 3.30; Br, 15.40. M.p. (°C, both enantiomers): 150. ^1^H NMR (CDCl_3_, 300 K, both enantiomers): δ 8.07 (d, 1H, *J*_HH_ = 7.4 Hz, BINOL), 7.96 (t, 2H, *J*_HH_ = 8.4 Hz, aromatic), 7.90–7.76 (m, 2H, aromatic), 7.64 (m, br, 2H, aromatic), 7.60–7.44 (m, 4H, aromatic), 7.42–7.28 (m, 5H, aromatic), 6.95 (d, 1H, *J*_HH_ = 8.8 Hz, BINOL). ^31^P{^1^H} NMR (CDCl_3_, 300 K, both enantiomers) δ 28.0 (FWHM = 90 Hz). IR (cm^−1^, both enantiomers): 3060–2970 m/w (aromatic ν_C-H_), 1620–1440 m (aromatic ν_C-C_), 1325 m (ν_C-O_), 1280–1240 m (ν_P=O_).

*Characterization of [MnCl_2_{O=PPh(BINOL)}_2_].* Anal. Calcd for C_52_H_34_Cl_2_MnO_6_P_2_ (942.61 g mol^−1^, %): C, 66.26; H, 3.64; Cl, 7.52. Found for (*R*,*R*)-[MnCl_2_{O=PPh(BINOL)}_2_] (%): C, 65.99; H, 3.67; Cl, 7.49. Found for (*S*,*S*)-[MnCl_2_{O=PPh(BINOL)}_2_] (%): C, 66.29; H, 3.65; Cl, 7.48. M.p. (°C, both enantiomers): 160. χ_M_^corr^ (c.g.s.u., both enantiomers): 1.39·10^−2^. ^31^P{^1^H} NMR (CDCl_3_, 300 K, both enantiomers) δ 27.2 (FWHM = 200 Hz). IR (cm^−1^, both enantiomers): 3057 w (aromatic ν_C-H_), 1620–1439 w (aromatic ν_C-C_), 1326 m (ν_C-O_), 1258 m (ν_P=O_).

*Characterization of [MnBr_2_{O=PPh(BINOL)}_2_].* Anal. Calcd for C_52_H_34_Br_2_MnO_6_P_2_ (1031.52 g mol^−1^, %): C, 60.55; H, 3.32; Br, 15.49. Found for (*R*,*R*)-[MnBr_2_{O=PPh(BINOL)}_2_] (%): C, 60.31; H, 3.33; Br, 15.43. Found for (*S*,*S*)-[MnBr_2_{O=PPh(BINOL)}_2_] (%): C, 60.35; H, 3.33; Br, 15.45. M.p. (°C, both enantiomers): 150. χ_M_^corr^ (c.g.s.u., both enantiomers): 1.45·10^−2^. ^31^P{^1^H} NMR (CDCl_3_, 300 K, both enantiomers) δ around 28 (FWHM ≈ 5000 Hz). IR (cm^−1^, both enantiomers): 3057 w (aromatic ν_C-H_), 1620–1439 w (aromatic ν_C-C_), 1325 m (ν_C-O_), 1256 m (ν_P=O_).

### 3.4. Optical Measurements

Absorption spectra were recorded with a Yoke 6000Plus double-beam spectrophotometer (Fengxian, China). Specific rotation [α]^20^_D_ was measured for acetone solutions using an Exacta+Optech (Modena, Italy) Polarimeter model PL1 LED (λ = 589.3 nm).

Photoluminescence emission (PL) and excitation (PLE) spectra as well as lifetime decay curves were registered on solid samples at room temperature using a Horiba Jobin Yvon (Kyoto, Japan) Fluorolog-3 spectrofluorometer. Air-tight quartz sample holders were used and filled in the glove box to avoid interactions of the air-sensible complexes with moisture. A continuous wave xenon arc lamp was used as the source, and the excitation wavelength was selected using a double Czerny–Turner monochromator. Suitable long-pass filters were placed in front of the acquisition systems. The detector was composed of a single Horiba (Kyoto, Japan) iHR 320 monochromator and a Hamamatsu (Shizuoka, Japan) R928 photomultiplier tube. The excitation and emission spectra were corrected for the instrumental functions. Time-resolved analyses were performed in multi-channel scaling mode (MCS) or time-correlated single photon counting mode (TCSPC) employing Horiba SpectraLED and NanoLED pulsed sources. The room-temperature photoluminescence quantum yields (Φ) at the solid state were measured employing an OceanOptics (Orlando, FL, USA) HR4000CG UV–vis–NIR detector, fiber-coupled to an integrating sphere connected to OceanOptics UV LED continuous sources. The values are reported as the average of three measurements.

### 3.5. Crystal Structure Determination

The crystallographic data were collected at CACTI (Universidade de Vigo) at 100 K (CryoStream 800) using a Bruker D8 Venture Photon II CMOS detector (Billerica, MA, USA) and Mo-Kα radiation (λ = 0.71073 Å) generated by an Incoatec high brilliance IμS microsource (Geesthacht, Germany). APEX3 version 2019-11-0 [56] was used for collecting frames of data, indexing reflections, and the determination of lattice parameters; SAINT version 8.40B [56] for integration of the intensity of reflections; and SADABS version 2016/2 [56] for scaling and empirical absorption correction. The crystallographic treatment was performed using the Oscail program [57] and solved using the SHELXT version 2018/2 program [58]. The structure was subsequently refined by full-matrix least-squares methods based on *F*^2^ using the SHELXL version 2018/3 program [59]. Non-hydrogen atoms were refined with anisotropic displacement parameters. Hydrogen atoms were calculated on the basis of a riding model and refined with isotropic displacement parameters. Other details concerning crystal data and structural refinement are given in Supplementary Materials Table S1. CCDC 2298339 contains the supplementary crystallographic data for this paper. These data can be obtained free of charge from the Cambridge Crystallographic Data Centre via https://www.ccdc.cam.ac.uk/data_request/cif (accessed on 17 December 2023). PLATON (version 140423) [60] was used to obtain some geometrical parameters from the CIF file.

### 3.6. Computational Details

Geometry optimizations and TD-DFT calculations [61] were carried out without symmetry constraints using the r2-SCAN-3c method [62], based on the meta-GGA r^2^SCAN functional [63] combined with a tailor-made triple-ζ Gaussian atomic orbital basis set with refitted D4 and geometrical counter-poise corrections for London-dispersion and basis set superposition error [64,65,66]. Further calculations were performed with the GGA-based B97-3c method [67], particularly for the geometry optimization of excited singlet states. The C-PCM implicit solvation model was added to all the calculations, considering dichloromethane as a continuous medium [68,69]. Calculations were carried out using ORCA 5.0.3 [70,71], and the output files were analyzed with Multiwfn, version 3.8 [72]. The cartesian coordinates of the DFT-optimized structures are provided in Supplementary Materials Table S2.

## 4. Conclusions

The BINOL-based phosphonates here investigated were revealed to be suitable O-donor ligands for first-row d-block divalent metal centers such as Mn(II) and Zn(II). The Zn(II) complex exhibited noticeable photoluminescence centered in the near-UV and was attributed to fluorescence from excited states localized on the BINOL fragments.

The replacement of Zn(II) with Mn(II) causes a noticeable change in the emission features, an influence associated in primis with easier intersystem crossing processes. Luminescence in the red region with lifetimes in the hundreds of seconds range is superimposed on the ligand-centered fluorescence. The choice of the halide affects the ratios of the bands in the PL and PLE spectra, while the luminescence lifetimes are roughly the same, moving from the chloro- to the bromo-complex. Such an outcome suggests that the observed emissions at longer wavelengths could be attributed to ligand-centered excited states populated by intersystem crossing.

The chiroptical behavior of the new compounds requires further deepening planned for the future, for instance, by measuring the circular dichroism and the circularly polarized luminescence. Moreover, the research on BINOL-based phosphonate complexes will be prosecuted by trying to isolate other coordination compounds with O=PPh(BINOL) as ligand.

## Figures and Tables

**Table 1:** Luminescence data for the [MX_2_{O=PPh(BINOL)}_2_] complexes (M = Zn, X = Br; M = Mn, X = Cl, Br; both enantiomers). Solid samples, r.t.

Complex	PL Max (nm)	PLE (nm)	τ	Φ (%)
[ZnBr_2_{O=PPh(BINOL)}_2_]	370	320, <300	8 ns	81
[MnCl_2_{O=PPh(BINOL)}_2_]	380, 653	410–500, <400	220 μs (58%), 700 μs (42%)	48
[MnBr_2_{O=PPh(BINOL)}_2_]	380, 641	410–500, <400	216 μs (62%), 530 μs (38%)	14

## Data Availability

Data are contained within the article and Supplementary Materials.

## Supplementary Materials

The following supporting information can be downloaded at: https://www.mdpi.com/article/10.3390/molecules29010239/s1, Figure S1: ^1^H NMR spectrum of (*S*)-O=PPh(BINOL); Figure S2: ^31^P{^1^H} NMR spectrum of (*S*)-O=PPh(BINOL); Figure S3: ^13^C{^1^H} NMR spectrum of (*S*)-O=PPh(BINOL); Figure S4: ^1^H-^13^C HSQC NMR spectrum of (*S*)-O=PPh(BINOL); Figure S5: ^1^H NMR spectrum of (*S*,*S*)-[ZnBr_2_{O=PPh(BINOL)}_2_]; Figure S6: ^31^P{^1^H} NMR spectrum of (*S*,*S*)-[ZnBr_2_{O=PPh(BINOL)}_2_]; Figure S7: IR spectra of (*S*)-O=PPh(BINOL) and (*S*,*S*)-[ZnBr_2_{O=PPh(BINOL)}_2_]; Figure S8: TGA curves of the [MX_2_{O=PPh(BINOL)}_2_] complexes; Figure S9: PL spectrum of [Zn{O=PPh(BINOL)}_2_] in CH_2_Cl_2_; Figure S10: IR spectra of (*S*,*S*)-[MnCl_2_{O=PPh(BINOL)}_2_] and (*S*,*S*)-[MnBr_2_{O=PPh(BINOL)}_2_]; Figure S11: PL spectra of solid O=PPh(BINOL); Figure S12: DFT optimized geometries of [ZnBr_2_{O=PPh(BINOL)}_2_] and of [MnBr_2_{O=PPh(BINOL)}_2_] with Gibbs energy differences, hole and electron distributions, and spin density surfaces; Figure S13: DFT optimized geometries of [MnBr_2_{O=PPh(BINOL)}_2_] and [MnBr_2_{O=PPh(BINOL)}_2_]_2_ with Gibbs energy difference. Table S1: Crystal data and structure refinement for (*S*)-O=PPh(BINOL); Table S2: Cartesian coordinates of the DFT-optimized structures.
