# Supplementary material for: Luminescent Behavior of Zn(II) and Mn(II) Halide Derivatives of 4-Phenyldinaphtho[2,1-d:1′,2′-f][1,3,2]dioxaphosphepine 4-Oxide and Single-Crystal X-ray Structure Determination of the Ligand
Source: Molecules. 2024 Jan 1;29(1):239. doi: 10.3390/molecules29010239 (PMC10780406; doi:10.3390/molecules29010239)
Supplement: Supplementary file 1 [file molecules-29-00239-s001.zip › Supplementary Material.pdf]

Luminescent Behaviour of Zn(II) and Mn(II) Halide Derivatives of 4-Phenyldinaphtho[2,1-d:1',2'-f][1,3,2]dioxaphosphepine 4-Oxide and Single-Crystal X-ray Structure Determination of the Ligand

Valentina Ferraro [1], Jesús Castro [2], Marco Bortoluzzi [1,*]

[1] Dipartimento di Scienze Molecolari e Nanosistemi, Università Ca' Foscari Venezia, 30172 Mestre VE, Italy; valentina.ferraro@unive.it

[2] Departamento de Química Inorgánica, Facultade de Química, Edificio de Ciencias Experimentais, Universidade de Vigo, 36310 Vigo Galicia, Spain; jesusc@uvigo.gal

* Correspondence: markos@unive.it; Tel.: +39-0412348651

Supplementary Material

Selected NMR spectra of O=PPh(BINOL)	Figures S1-S4
Crystal data and structure refinement for (*S*)-O=PPh(BINOL)	Table S1
Selected NMR spectra of [Zn{O=PPh(BINOL)}$_2$]	Figures S5-S6
IR spectra of (*S*)-O=PPh(BINOL) and (*S*,*S*)- [ZnBr$_2${O=PPh(BINOL)}$_2$]	Figure S7
TGA curves of the [MX$_2${O=PPh(BINOL)}$_2$] complexes	Figure S8
PL spectrum of [Zn{O=PPh(BINOL)}$_2$] in CH_2Cl_2	Figure S9
IR spectra of (*S*,*S*)-[MnCl$_2${O=PPh(BINOL)}$_2$] and (*S*,*S*)-[MnBr$_2${O=PPh(BINOL)}$_2$]	Figure S10
PL spectra of O=PPh(BINOL)	Figure S11
DFT optimized geometries of [ZnBr$_2${O=PPh(BINOL)}$_2$] (singlet and triplet states) and of [MnBr$_2${O=PPh(BINOL)}$_2$] (sextet and octet states) with Gibbs energy differences and selected surfaces.	Figure S12
DFT optimized geometries of [MnBr$_2${O=PPh(BINOL)}$_2$] and [MnBr$_2${O=PPh(BINOL)}$_2$]$_2$ with Gibbs energy difference	Figure S13
Cartesian coordinates of the DFT-optimized structures	Table S2

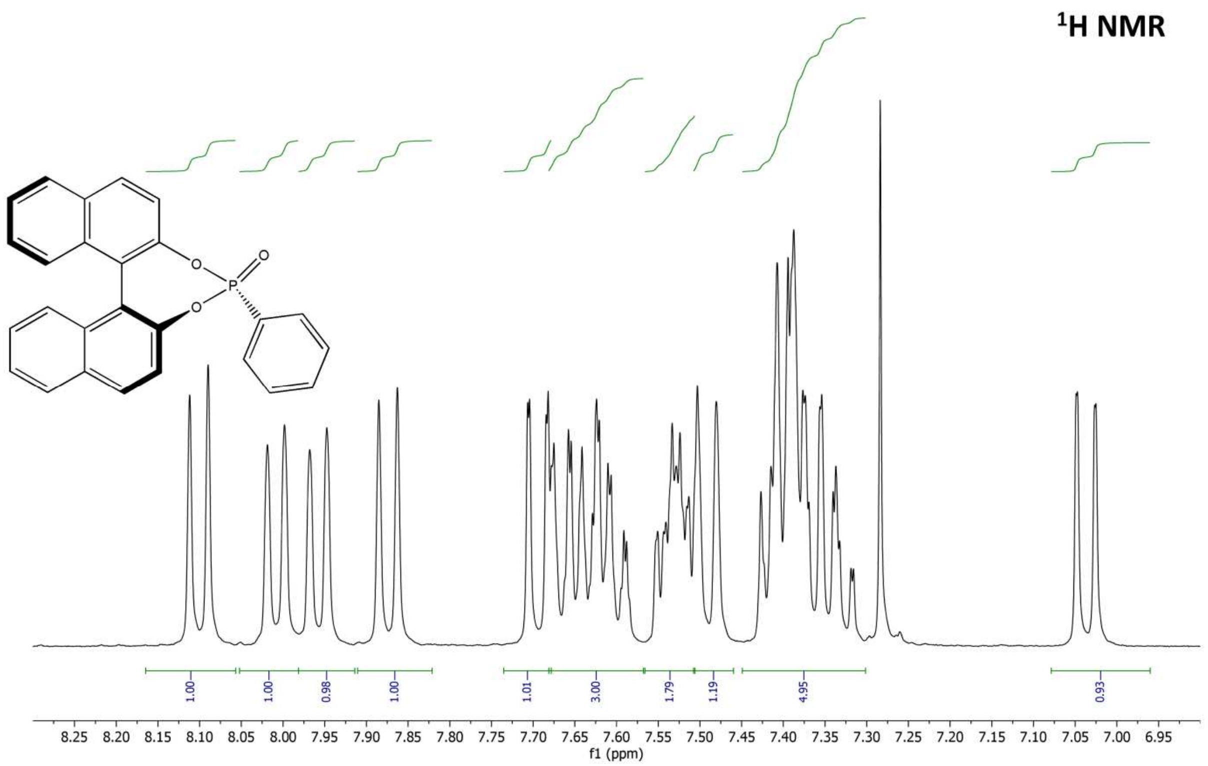

Figure S1. ^{1}H NMR spectrum of (*S*)-O=PPh(BINOL) ($CDCl_3$, 300 K).

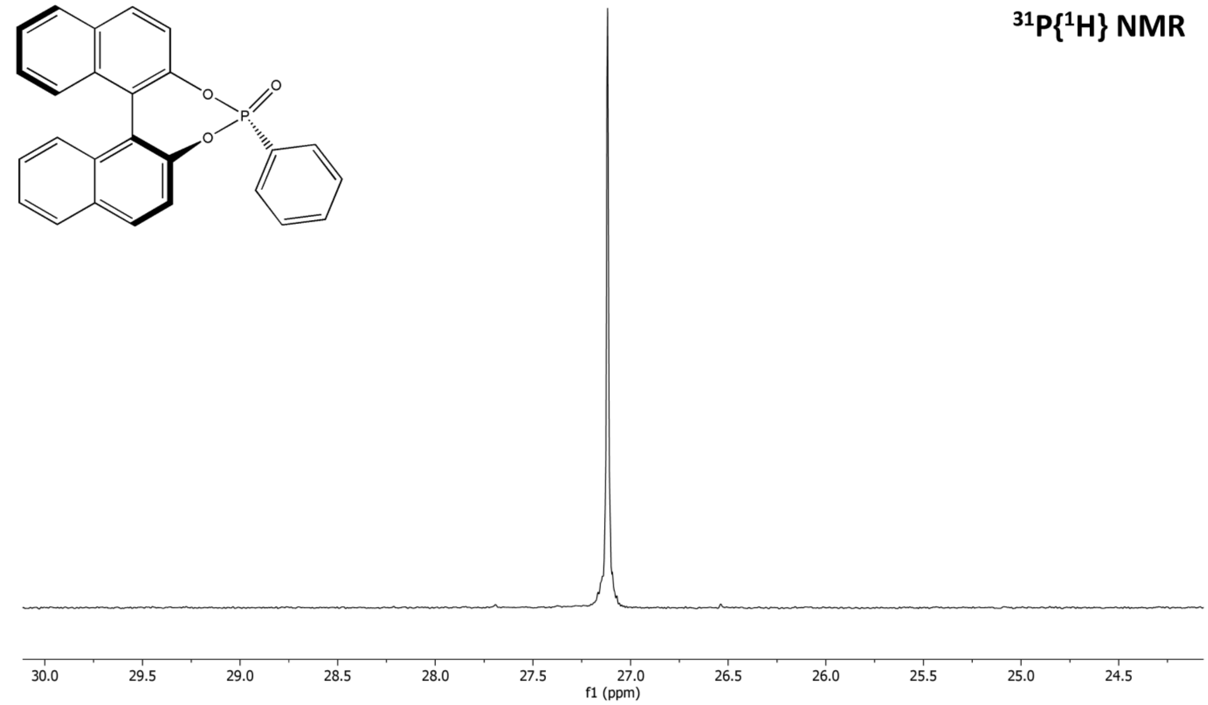

Figure S2. ^{31}P{^{1}H} NMR spectrum of (*S*)-O=PPh(BINOL) ($CDCl_3$, 300 K).

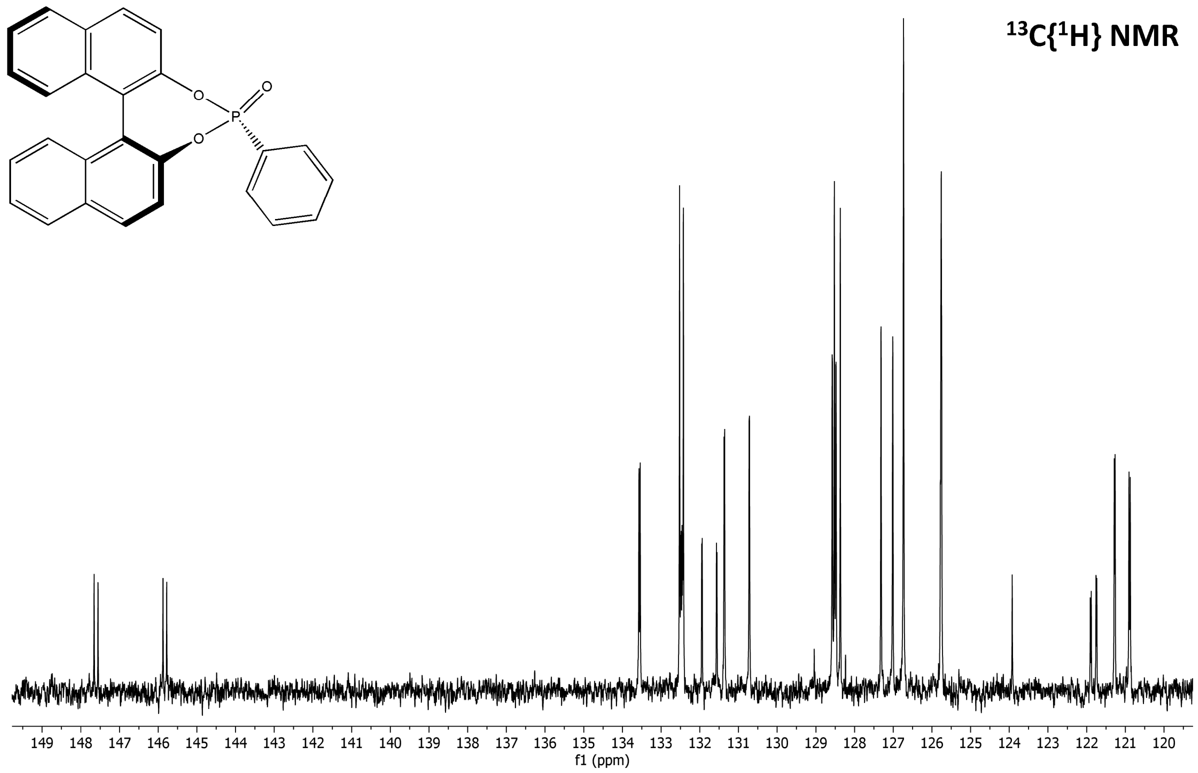

Figure S3. $^{13}C\{^{1}H\}$ NMR spectrum of (*S*)-O=PPh(BINOL) ($CDCl_3$, 300 K).

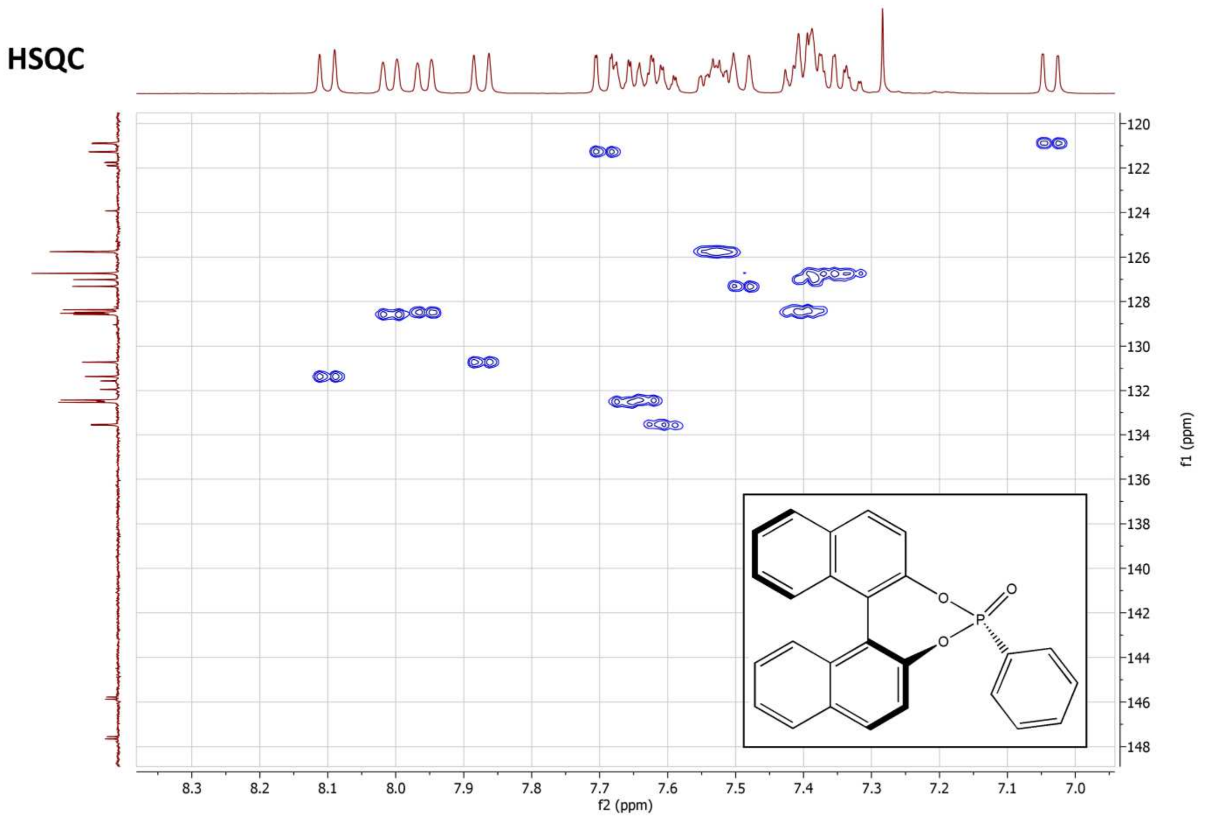

Figure S4. ^{1}H-^{13}C HSQC NMR spectrum of (*S*)-O=PPh(BINOL) ($CDCl_3$, 300 K).

Table S1. Crystal data and structure refinement for (*S*)-O=PPh(BINOL).

Empirical formula	C_{26} H_{17} O_3 P
Formula weight	408.36
Temperature	100(2) K
Wavelength	0.71073 Å
Crystal system	Orthorhombic
Space group	$P2_12_12_1$
Unit cell dimensions	a = 10.1725(3) Å
	b = 10.5825(3) Å
	c = 18.4246(5) Å
Volume	1983.42(10) Å^3
Z	4
Density (calculated)	1.368 Mg/m^3
Absorption coefficient	0.165 mm^{-1}
F(000)	848
Crystal size	0.208 x 0.177 x 0.148 mm
Theta range for data collection	2.211 to 28.315°
Index ranges	$-13 \le h \le 13$
	$-14 \le k \le 14$
	$-24 \le l \le 24$
Reflections collected	31587
Independent reflections	4932 [R_{int} = 0.0204]
Reflections observed (>2σ)	4866
Data Completeness	0.999
Absorption correction	Semi-empirical from equivalents
Max. and min. transmission	0.7457 and 0.6981
Refinement method	Full-matrix least-squares on F^2
Data / restraints / parameters	4932 / 0 / 271
Goodness-of-fit on F^2	1.091
Final *R* indices [I>2σ(I)]	R_1 = 0.0273
	wR_2 = 0.0731
R indices (all data)	R_1 = 0.0277
	wR_2 = 0.0734
Absolute structure parameter	-0.004(14)
Largest diff. peak and hole	0.348 and -0.311 e.Å^{-3}

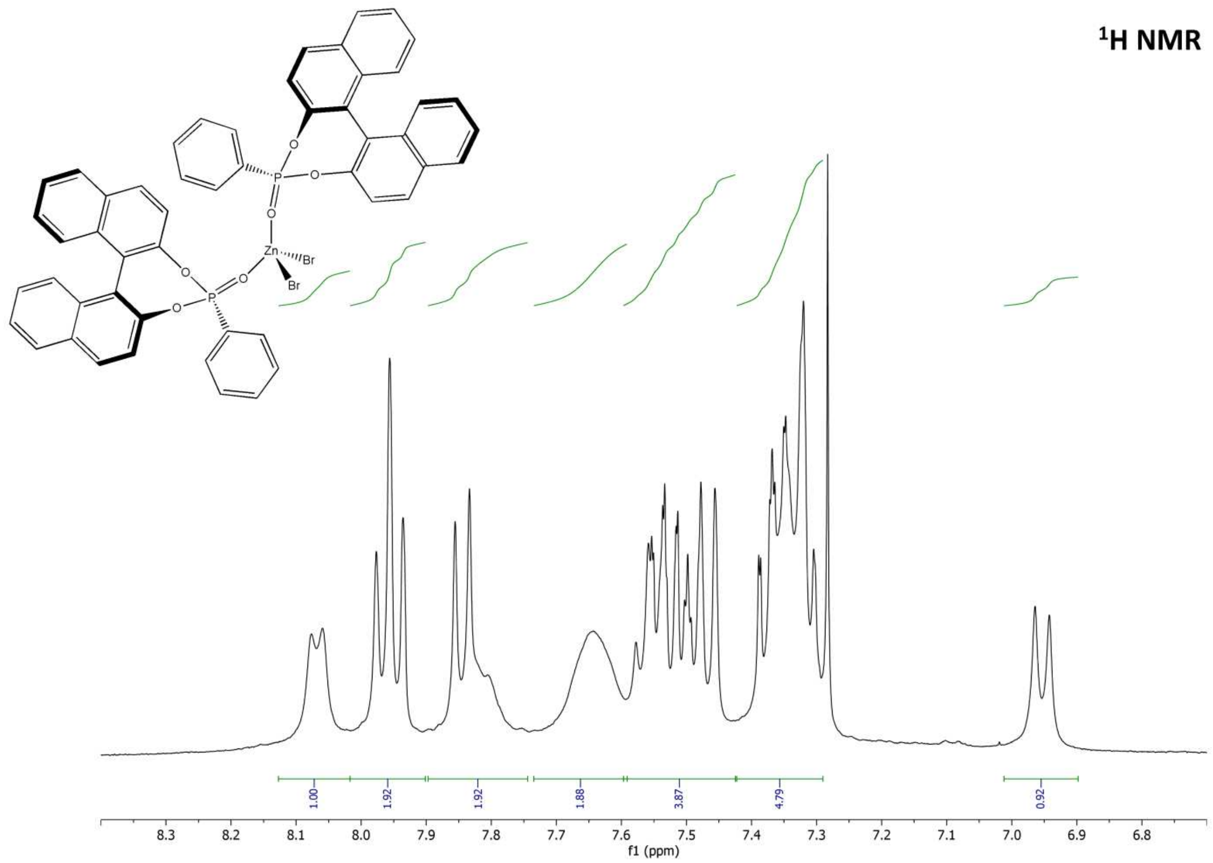

Figure S5. ^{1}H NMR spectrum of (*S*,*S*)-[$ZnBr_2${O=PPh(BINOL)}$_2$] ($CDCl_3$, 300 K).

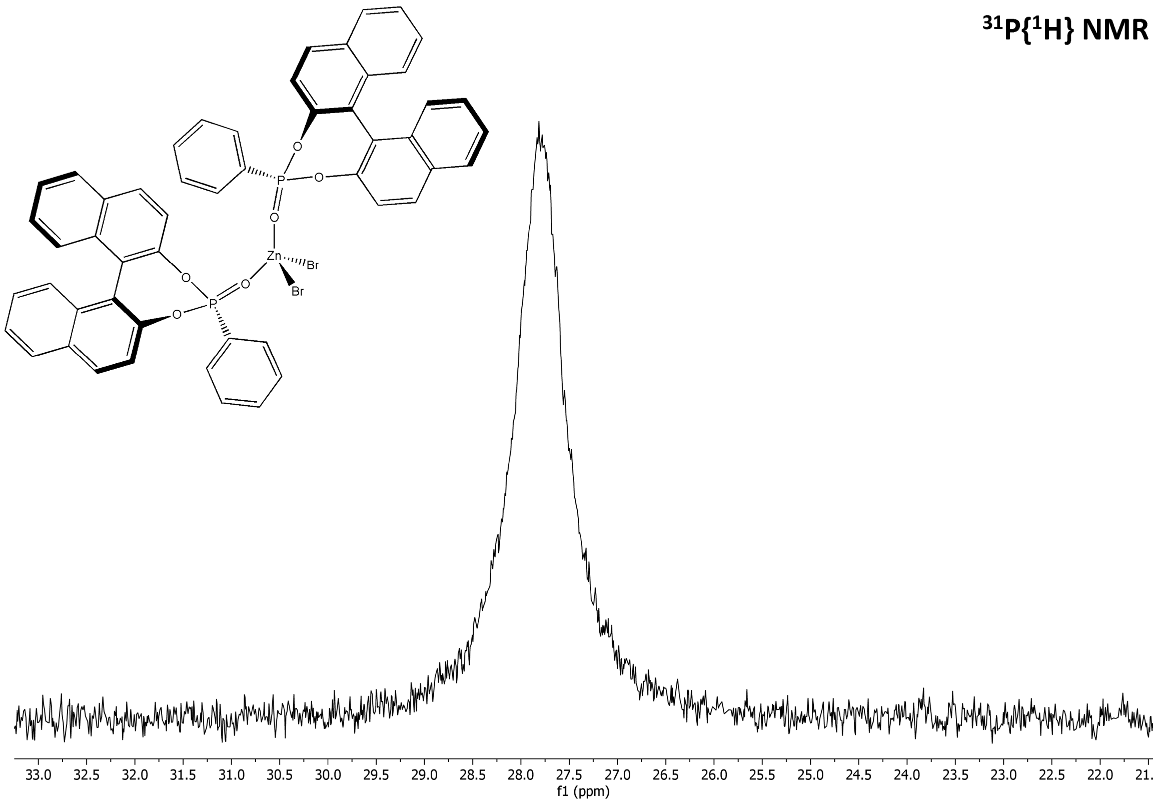

Figure S6. ^{31}P{^{1}H} NMR spectrum of (*S*,*S*)-[$ZnBr_2${O=PPh(BINOL)}$_2$] ($CDCl_3$, 300 K).

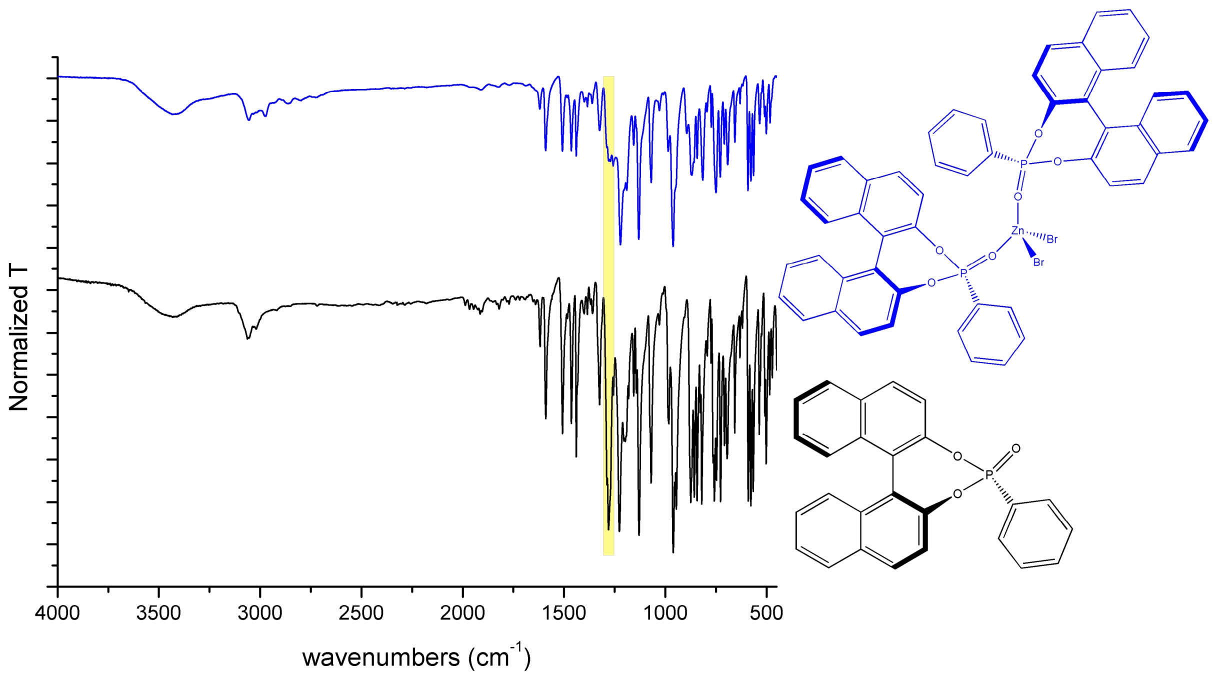

Figure S7. IR spectra (KBr) of (*S*)-O=PPh(BINOL) and (*S*,*S*)-[$ZnBr_2${O=PPh(BINOL)}$_2$].

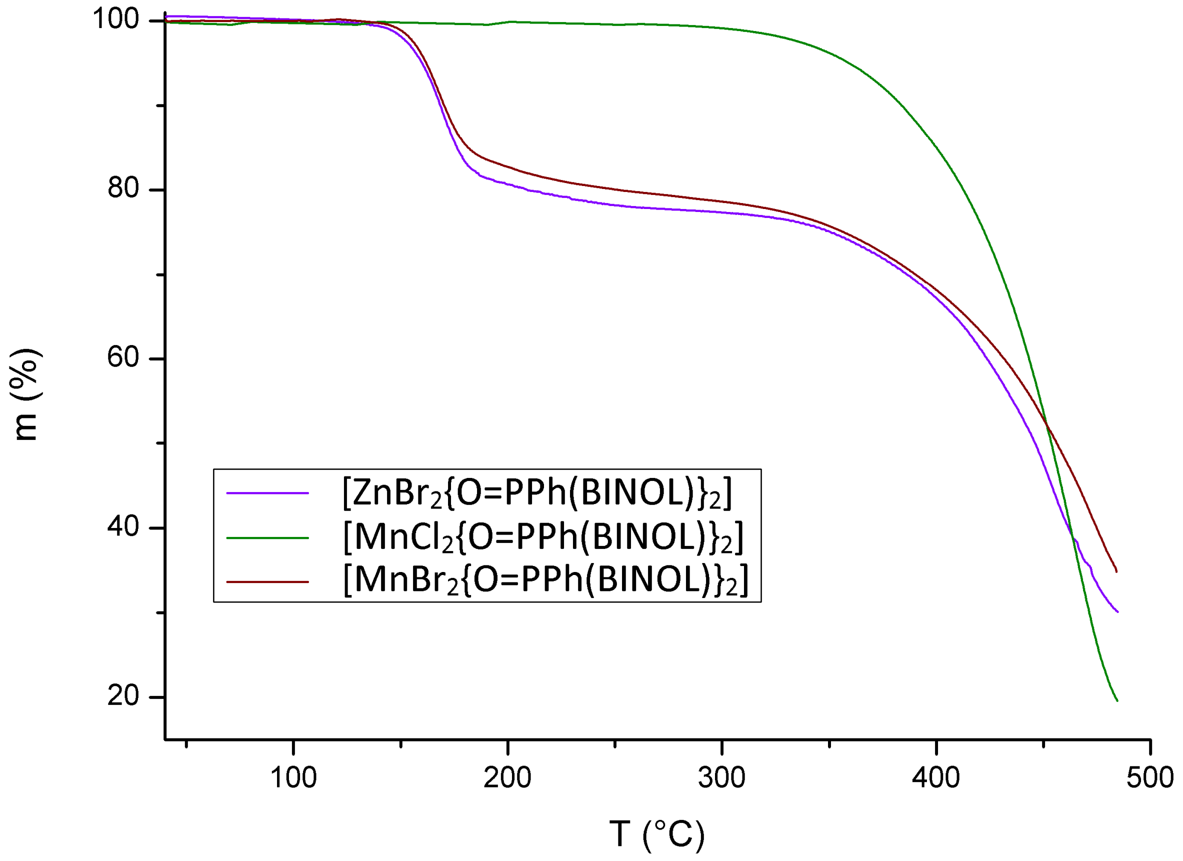

Figure S8. TGA curves of the [MX_2{O=PPh(BINOL)}$_2$] complexes (M = Zn, X = Br; M = Mn, X = Cl, Br), *S*,*S*-enantiomers.

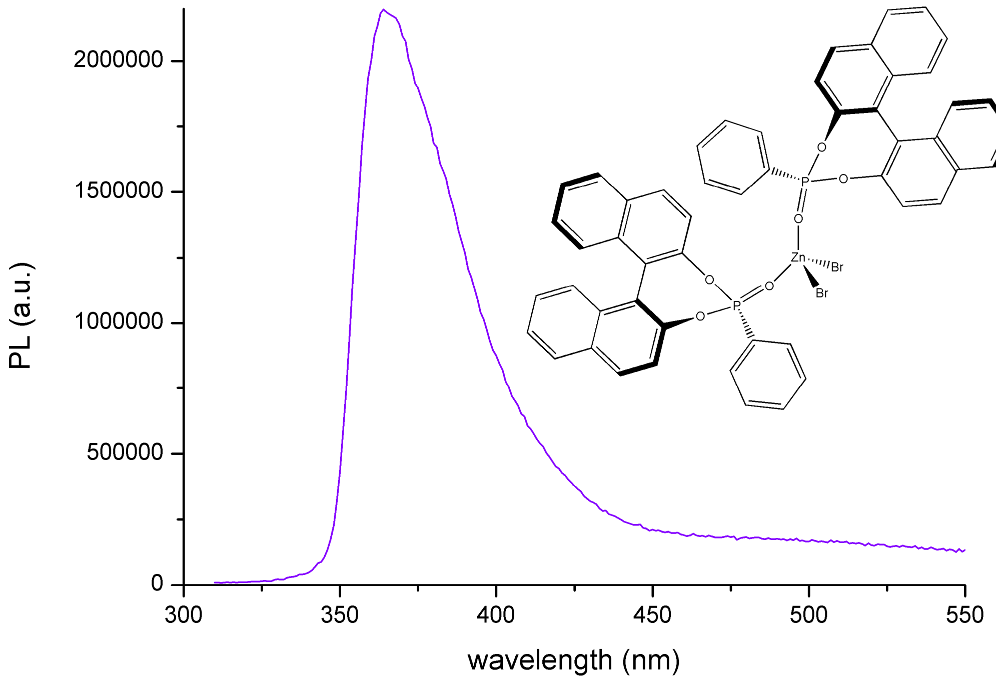

Figure S9. PL spectrum ($\lambda_{excitation}$ = 280 nm, r.t.) of (*S*,*S*)-[$ZnBr_2${O=PPh(BINOL)}$_2$], $6{\cdot}10^{-3}$ M in CH_2Cl_2.

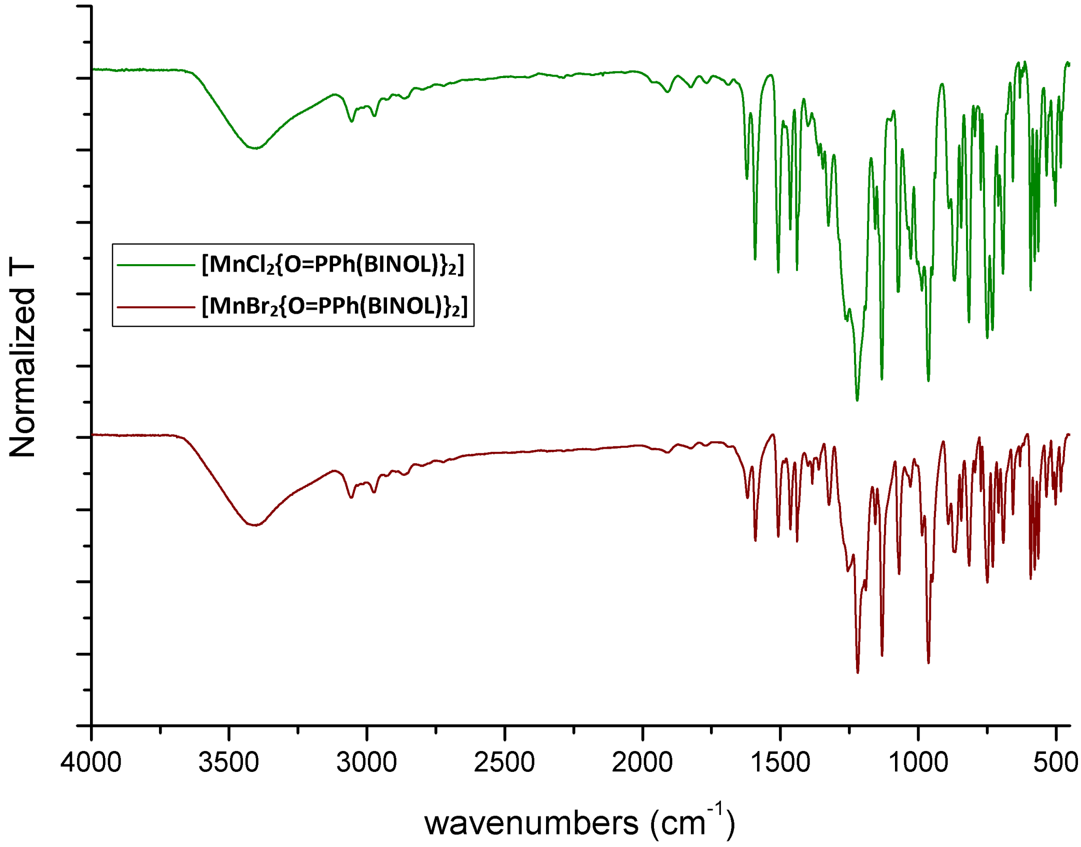

Figure S10. IR spectra (KBr) of (*S*,*S*)-[$MnCl_2${O=PPh(BINOL)}$_2$] and (*S*,*S*)-[$MnBr_2${O=PPh(BINOL)}$_2$].

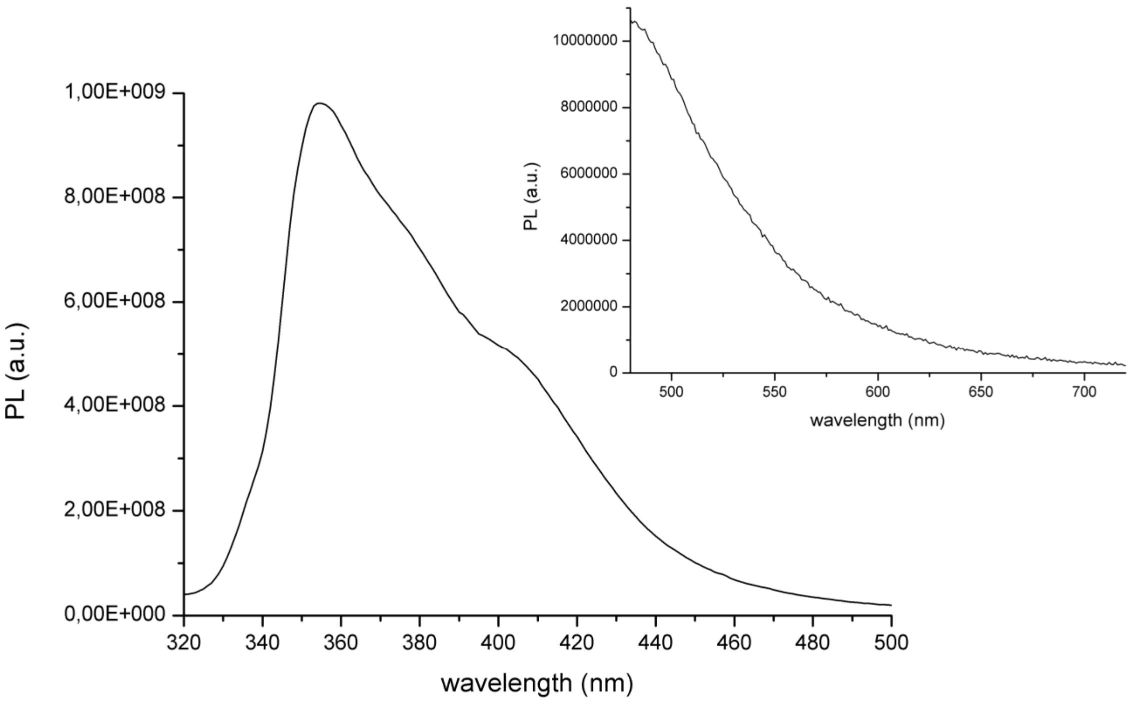

Figure S11. PL spectra (r.t.) of solid O=PPh(BINOL).

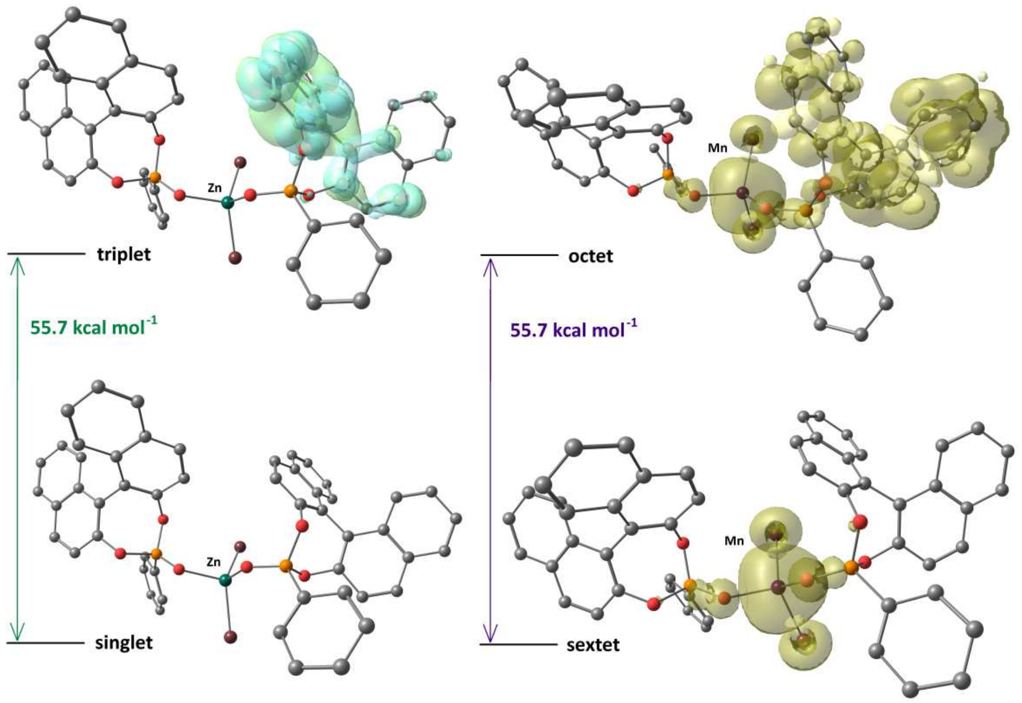

Figure S12. DFT optimized (C-PCM/r^{2}SCAN-3c) geometries of [$ZnBr_2${O=PPh(BINOL)}$_2$] (singlet and triplet states) and of [$MnBr_2${O=PPh(BINOL)}$_2$] (sextet and octet states) with Gibbs energy differences. Colour map: Zn, green; Mn, violet; Br, dark red; O, red; P, orange; C, grey. Hydrogen atoms are omitted for clarity. Hole (light blue) and electron (green) distributions for the [$ZnBr_2${O=PPh(BINOL)}$_2$] $T_1 \leftarrow S_0$ transition. Phosphorescence is the reverse process. Spin density surfaces (yellow tones) of [$MnBr_2${O=PPh(BINOL)}$_2$]. Surfaces isovalue = 0.001 a.u.

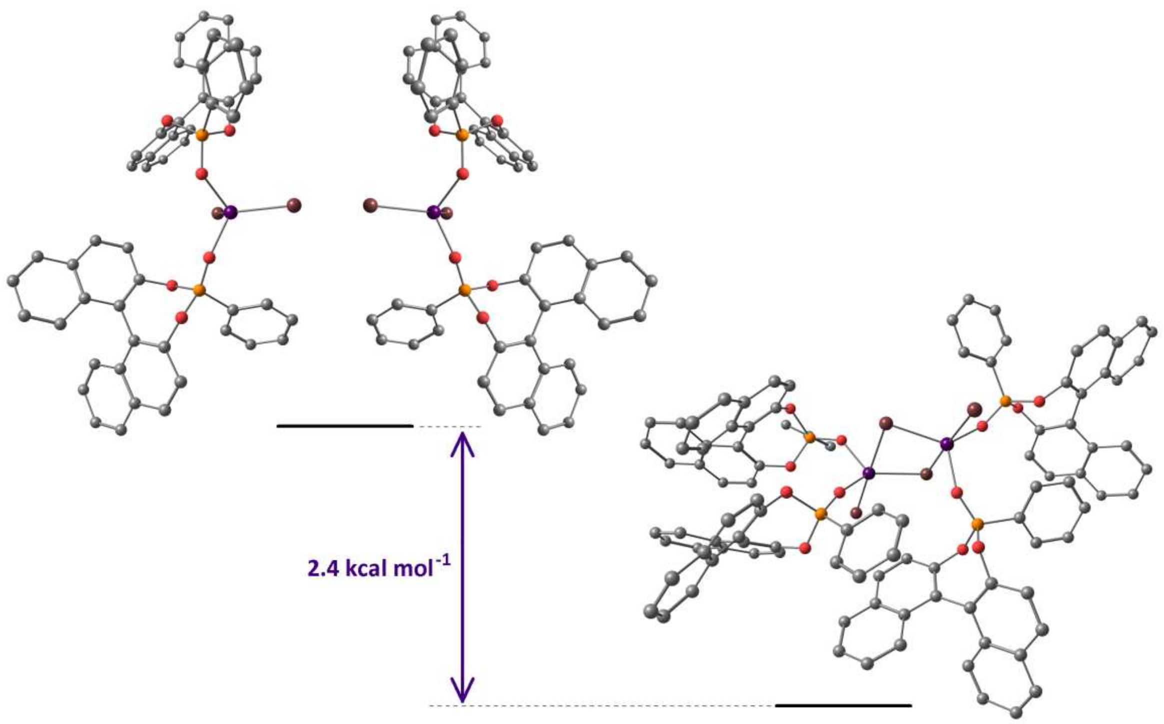

Figure S13. DFT optimized (C-PCM/r^{2}SCAN-3c) geometries of [$MnBr_2${O=PPh(BINOL)}$_2$] (sextet state) and [$MnBr_2${O=PPh(BINOL)}$_2$]$_2$ (undecet state) with Gibbs energy difference. Colour map: Mn, violet; Br, dark red; O, red; P, orange; C, grey. Hydrogen atoms are omitted for clarity.

Table S2. Cartesian coordinates of the DFT-optimized structures.

[$ZnBr_2${O=PPh(BINOL)}$_2$] (singlet state, r^2SCAN-3c)				[$ZnBr_2${O=PPh(BINOL)}$_2$] (triplet state, r^2SCAN-3c)			
H	-4.856730000	-0.073988000	5.281349000	H	-4.959528000	-0.230136000	5.422785000
C	-3.812513000	0.196178000	5.160556000	C	-3.926985000	0.046459000	5.234989000
H	-4.104354000	2.307371000	5.309819000	H	-4.223951000	2.183417000	5.548590000
C	-3.389734000	1.500151000	5.170674000	C	-3.502454000	1.406407000	5.316279000
C	-1.509999000	-0.594845000	4.851019000	C	-1.600561000	-0.647539000	4.763655000
C	-2.026007000	1.817530000	4.961602000	C	-2.163433000	1.765274000	5.008895000
C	-2.863263000	-0.819542000	4.986093000	C	-3.023699000	-0.913349000	4.932573000
C	-1.071172000	0.765321000	4.781019000	C	-1.207633000	0.743700000	4.687292000
C	-1.592753000	3.164392000	4.897120000	C	-1.760177000	3.108389000	4.963435000
H	0.995393000	0.330388000	4.300166000	H	0.761803000	0.372995000	3.915907000
C	-0.279624000	3.468556000	4.635104000	C	-0.456300000	3.470162000	4.564843000
H	-2.326528000	3.952819000	5.044401000	H	-2.477795000	3.878597000	5.233697000
H	0.041518000	4.504672000	4.580510000	H	-0.172130000	4.518075000	4.544486000
C	0.653000000	2.433066000	4.414405000	C	0.437670000	2.493429000	4.189605000
H	1.683866000	2.680109000	4.177274000	H	1.438037000	2.753699000	3.858002000
C	0.269208000	1.115005000	4.485440000	C	0.054382000	1.132031000	4.235488000
C	-0.782276000	-2.704013000	3.774252000	C	-0.860564000	-2.796878000	3.795076000
C	-0.579446000	-1.740522000	4.741363000	C	-0.657421000	-1.740649000	4.689854000
C	1.173109000	-3.936072000	4.378440000	C	1.221024000	-3.853087000	4.322637000
C	0.517536000	-1.925230000	5.640910000	C	0.531715000	-1.807949000	5.510488000
C	0.077937000	-3.792721000	3.566413000	C	0.053674000	-3.829526000	3.592369000
C	1.410005000	-3.027127000	5.437793000	C	1.478047000	-2.859383000	5.299059000
C	0.731997000	-1.083267000	6.760449000	C	0.757573000	-0.913437000	6.580159000
H	-0.143209000	-4.493295000	2.767392000	H	-0.164543000	-4.580323000	2.839176000
H	3.179385000	-4.038470000	6.146734000	H	3.360567000	-3.712545000	5.916503000
H	1.859145000	-4.765874000	4.231381000	H	1.948265000	-4.644900000	4.167073000
C	1.794814000	-1.293311000	7.606505000	C	1.893703000	-1.002565000	7.353873000
H	0.040650000	-0.269873000	6.954093000	H	0.020422000	-0.147855000	6.798033000
H	1.936903000	-0.640170000	8.462814000	H	2.042162000	-0.303207000	8.171695000
C	2.698670000	-2.352828000	7.379627000	C	2.856516000	-1.998995000	7.102653000
H	3.537824000	-2.500677000	8.053167000	H	3.752248000	-2.054576000	7.714441000
C	2.503902000	-3.204548000	6.320247000	C	2.643739000	-2.916270000	6.100981000
O	-3.341592000	-2.145825000	4.966809000	O	-3.447139000	-2.253334000	4.935649000
O	-1.887415000	-2.591229000	2.917212000	O	-1.968174000	-2.764979000	2.935175000
P	-3.360055000	-2.866418000	3.526787000	P	-3.446091000	-3.029058000	3.525951000
O	-4.370618000	-2.327044000	2.556272000	O	-4.446891000	-2.535601000	2.521460000
C	-3.553781000	-4.588782000	3.920860000	C	-3.632834000	-4.735047000	3.982036000
C	-3.750420000	-7.314008000	4.396021000	C	-3.813600000	-7.440635000	4.563926000
C	-4.225388000	-5.404598000	3.002959000	C	-4.317763000	-5.586568000	3.107367000
C	-2.975693000	-5.132640000	5.075164000	C	-3.033288000	-5.233454000	5.146231000
C	-3.080426000	-6.498776000	5.306714000	C	-3.130092000	-6.590129000	5.431187000
C	-4.321760000	-6.769173000	3.248069000	C	-4.406433000	-6.940846000	3.406187000
H	-4.667470000	-4.972934000	2.109509000	H	-4.776569000	-5.189845000	2.206344000
H	-2.455528000	-4.497204000	5.785637000	H	-2.503674000	-4.570430000	5.823940000
H	-2.639128000	-6.927403000	6.201324000	H	-2.672292000	-6.983543000	6.333671000
H	-4.845557000	-7.406626000	2.542372000	H	-4.940433000	-7.605968000	2.734514000
H	-3.829352000	-8.381044000	4.583953000	H	-3.886290000	-8.499943000	4.793477000
H	-5.540897000	1.444672000	3.019184000	H	-5.588654000	1.268256000	3.150405000
C	-6.618045000	1.580077000	3.042921000	C	-6.660948000	1.439973000	3.139376000
H	-6.793016000	1.679665000	5.170213000	H	-6.907682000	1.501754000	5.261116000
C	-7.314772000	1.704839000	4.217009000	C	-7.393905000	1.565362000	4.291085000
C	-8.685892000	1.824884000	1.741317000	C	-8.670753000	1.789291000	1.772379000
C	-8.724790000	1.835113000	4.210292000	C	-8.797176000	1.746714000	4.238369000
C	-7.323308000	1.633806000	1.832702000	C	-7.319991000	1.544783000	1.906387000
C	-9.428769000	1.874602000	2.963579000	C	-9.454437000	1.838193000	2.969077000
C	-9.457192000	1.897696000	5.421123000	C	-9.569343000	1.810262000	5.424274000
H	-11.394713000	1.899950000	2.055335000	H	-11.385924000	1.951666000	1.994842000
C	-10.828798000	1.964516000	5.411125000	C	-10.936501000	1.926291000	5.368368000
H	-8.909710000	1.877743000	6.360101000	H	-9.056397000	1.750763000	6.380923000
H	-11.381284000	2.006351000	6.345290000	H	-11.519911000	1.968178000	6.283538000
C	-11.525341000	1.957835000	4.184085000	C	-11.588814000	1.969833000	4.118072000
H	-12.611401000	1.980477000	4.182599000	H	-12.672705000	2.030687000	4.079285000
C	-10.844766000	1.914289000	2.990509000	C	-10.868321000	1.927485000	2.948135000
C	-9.130929000	0.935989000	-0.527558000	C	-9.068174000	0.975345000	-0.533063000
C	-9.327881000	1.928197000	0.411862000	C	-9.261835000	1.949929000	0.425118000
C	-10.567392000	1.966101000	-2.135325000	C	-10.409867000	2.097006000	-2.160912000
C	-10.122023000	3.055456000	0.028166000	C	-10.001319000	3.114893000	0.044435000

C	-9.743891000	0.926175000	-1.789573000	C	-9.636412000	1.019603000	-1.815032000
C	-10.761127000	3.056618000	-1.253585000	C	-10.595066000	3.171386000	-1.257797000
C	-10.262015000	4.200822000	0.850731000	C	-10.128582000	4.244095000	0.891029000
H	-9.550868000	0.096841000	-2.462700000	H	-9.449194000	0.201092000	-2.502894000
H	-12.045884000	4.148396000	-2.600906000	H	-11.791771000	4.342523000	-2.619337000
H	-11.060095000	1.976011000	-3.103786000	H	-10.867777000	2.149229000	-3.144957000
C	-11.028812000	5.268105000	0.447776000	C	-10.842095000	5.348498000	0.489964000
H	-9.747551000	4.236186000	1.805234000	H	-9.646563000	4.237933000	1.862953000
H	-11.116988000	6.139263000	1.090672000	H	-10.921022000	6.206217000	1.151852000
C	-11.695409000	5.248223000	-0.795652000	C	-11.465428000	5.383902000	-0.775292000
H	-12.303466000	6.096545000	-1.096132000	H	-12.031738000	6.261204000	-1.074148000
C	-11.557065000	4.166383000	-1.630042000	C	-11.336917000	4.318624000	-1.632222000
O	-6.570340000	1.517449000	0.650711000	O	-6.528419000	1.430073000	0.750613000
O	-8.292301000	-0.147264000	-0.215820000	O	-8.278789000	-0.144511000	-0.222064000
P	-6.700395000	0.146337000	-0.181391000	P	-6.680002000	0.096256000	-0.136842000
O	-6.076607000	-1.076121000	0.430066000	O	-6.109523000	-1.168067000	0.441454000
C	-6.105161000	0.595313000	-1.796946000	C	-6.032061000	0.588116000	-1.719764000
C	-5.244254000	1.207436000	-4.362351000	C	-5.090180000	1.271087000	-4.238513000
C	-5.503910000	-0.395772000	-2.580945000	C	-5.464190000	-0.395416000	-2.537517000
C	-6.280719000	1.893075000	-2.294567000	C	-6.134669000	1.913654000	-2.160900000
C	-5.847100000	2.191356000	-3.580520000	C	-5.660596000	2.247459000	-3.423698000
C	-5.072475000	-0.082104000	-3.864081000	C	-4.991893000	-0.046529000	-3.796871000
H	-5.361942000	-1.398190000	-2.186383000	H	-5.379023000	-1.420456000	-2.187064000
H	-6.743669000	2.661772000	-1.683462000	H	-6.572822000	2.675639000	-1.523790000
H	-5.975519000	3.196426000	-3.970649000	H	-5.732077000	3.274073000	-3.769851000
H	-4.596817000	-0.845484000	-4.472256000	H	-4.541785000	-0.804612000	-4.430582000
H	-4.903146000	1.449149000	-5.365041000	H	-4.717056000	1.540365000	-5.222606000
Zn	-4.117461000	-1.520389000	0.715288000	Zn	-4.160021000	-1.678017000	0.709312000
Br	-3.503792000	-3.307606000	-0.747786000	Br	-3.590640000	-3.438159000	-0.799867000
Br	-2.901867000	0.532095000	0.880239000	Br	-2.902333000	0.342773000	0.929977000
**[$ZnBr_2${O=PPh(BINOL)}$_2$] (singlet state, B97-3c)**				**[$ZnBr_2${O=PPh(BINOL)}$_2$] (S_1 singlet state, B97-3c)**			
H	-4.883908000	-0.125489000	5.340024000	H	-4.881060000	-0.081611000	5.417824000
C	-3.852320000	0.152761000	5.184875000	C	-3.841888000	0.160611000	5.216275000
H	-4.165682000	2.252128000	5.332090000	H	-4.075015000	2.278929000	5.449290000
C	-3.447747000	1.458541000	5.174405000	C	-3.380920000	1.467230000	5.240186000
C	-1.560858000	-0.610708000	4.805668000	C	-1.571138000	-0.655602000	4.739789000
C	-2.101669000	1.792368000	4.915154000	C	-2.032116000	1.771189000	4.939519000
C	-2.903671000	-0.853206000	4.985129000	C	-2.955295000	-0.869759000	4.920855000
C	-1.143127000	0.751210000	4.708737000	C	-1.111518000	0.708104000	4.667766000
C	-1.688192000	3.138894000	4.822421000	C	-1.597297000	3.114041000	4.843646000
H	0.902438000	0.345040000	4.158235000	H	0.882555000	0.253640000	3.953725000
C	-0.393141000	3.456959000	4.510473000	C	-0.299626000	3.409184000	4.454882000
H	-2.421872000	3.917070000	4.988185000	H	-2.306382000	3.911322000	5.058961000
H	-0.089476000	4.492218000	4.434282000	H	0.024553000	4.444641000	4.375282000
C	0.540286000	2.434432000	4.266057000	C	0.587193000	2.368423000	4.137691000
H	1.554524000	2.690235000	3.990712000	H	1.595831000	2.599681000	3.801247000
C	0.175857000	1.116416000	4.362869000	C	0.189292000	1.041133000	4.235991000
C	-0.814701000	-2.716247000	3.737815000	C	-0.888559000	-2.835831000	3.759327000
C	-0.614082000	-1.737351000	4.686688000	C	-0.658860000	-1.791653000	4.677564000
C	1.180797000	-3.887373000	4.318032000	C	1.199864000	-3.895912000	4.284782000
C	0.503594000	-1.882351000	5.563228000	C	0.508234000	-1.882076000	5.522083000
C	0.066517000	-3.783142000	3.532611000	C	0.037100000	-3.857360000	3.536371000
C	1.419694000	-2.961206000	5.355565000	C	1.452038000	-2.937731000	5.297416000
C	0.721702000	-1.024947000	6.664274000	C	0.715853000	-1.023303000	6.624788000
H	-0.154428000	-4.500138000	2.755680000	H	-0.173004000	-4.597091000	2.768439000
H	3.225050000	-3.909061000	6.036559000	H	3.308741000	-3.842957000	5.934341000
H	1.878789000	-4.699894000	4.166914000	H	1.923756000	-4.691624000	4.117986000
C	1.805923000	-1.192616000	7.486126000	C	1.840500000	-1.153886000	7.424599000
H	0.017812000	-0.231881000	6.864244000	H	-0.018689000	-0.258356000	6.860367000
H	1.948780000	-0.526515000	8.326331000	H	1.978925000	-0.477394000	8.265865000
C	2.730000000	-2.226328000	7.253048000	C	2.795247000	-2.154050000	7.167532000
H	3.584485000	-2.342233000	7.905904000	H	3.675232000	-2.242204000	7.801585000
C	2.533827000	-3.095057000	6.212592000	C	2.597473000	-3.040616000	6.123931000
O	-3.370804000	-2.180899000	4.978394000	O	-3.460642000	-2.164107000	4.938513000
O	-1.941362000	-2.651952000	2.908774000	O	-2.002811000	-2.809477000	2.936966000
P	-3.404882000	-2.933636000	3.549413000	P	-3.511552000	-3.021401000	3.544447000
O	-4.439888000	-2.429943000	2.584504000	O	-4.504062000	-2.546079000	2.522815000
C	-3.563603000	-4.649024000	3.980272000	C	-3.717897000	-4.699252000	4.100716000
C	-3.706789000	-7.360251000	4.515277000	C	-3.957395000	-7.365507000	4.843992000
C	-4.234069000	-5.493664000	3.094161000	C	-4.429720000	-5.585390000	3.276412000
C	-2.959122000	-5.158451000	5.131288000	C	-3.118808000	-5.141112000	5.291868000

C	-3.036705000	-6.516729000	5.393088000	C	-3.245969000	-6.479171000	5.658127000
C	-4.303377000	-6.849861000	3.368978000	C	-4.546560000	-6.920530000	3.657459000
H	-4.694577000	-5.091292000	2.203050000	H	-4.886090000	-5.230554000	2.354462000
H	-2.439128000	-4.503123000	5.814595000	H	-2.569051000	-4.450006000	5.927399000
H	-2.574699000	-6.916525000	6.284845000	H	-2.791470000	-6.829926000	6.582793000
H	-4.824892000	-7.507811000	2.688193000	H	-5.101033000	-7.613416000	3.027574000
H	-3.764335000	-8.419535000	4.725870000	H	-4.053743000	-8.409822000	5.137332000
H	-5.608009000	1.330212000	3.106052000	H	-5.544746000	1.230724000	3.016152000
C	-6.675420000	1.491256000	3.102997000	C	-6.620441000	1.393009000	3.030859000
H	-6.898668000	1.566131000	5.217897000	H	-6.815912000	1.490221000	5.179717000
C	-7.395518000	1.616335000	4.258396000	C	-7.326708000	1.535639000	4.219340000
C	-8.695391000	1.796334000	1.756526000	C	-8.670270000	1.746506000	1.684282000
C	-8.797362000	1.776025000	4.220266000	C	-8.737550000	1.713008000	4.190406000
C	-7.341584000	1.575650000	1.877416000	C	-7.292029000	1.498932000	1.816259000
C	-9.466458000	1.844701000	2.957734000	C	-9.426443000	1.777504000	2.925393000
C	-9.558268000	1.838342000	5.407917000	C	-9.496762000	1.793517000	5.388365000
H	-11.402133000	1.920309000	2.009711000	H	-11.374889000	1.803365000	1.995168000
C	-10.923983000	1.932627000	5.365028000	C	-10.878300000	1.880152000	5.363781000
H	-9.039173000	1.795653000	6.356662000	H	-8.964977000	1.771677000	6.340645000
H	-11.496557000	1.973220000	6.281697000	H	-11.440309000	1.935814000	6.296086000
C	-11.586078000	1.954961000	4.124938000	C	-11.555719000	1.868793000	4.129646000
H	-12.666413000	1.999834000	4.095570000	H	-12.644810000	1.895795000	4.104395000
C	-10.876920000	1.912077000	2.952486000	C	-10.840281000	1.812063000	2.942119000
C	-9.116346000	0.964347000	-0.536106000	C	-9.055834000	1.005991000	-0.676230000
C	-9.303618000	1.940565000	0.419050000	C	-9.283943000	1.924774000	0.372566000
C	-10.492905000	2.070696000	-2.140600000	C	-10.573706000	2.099067000	-2.192176000
C	-10.057797000	3.095148000	0.047862000	C	-10.125223000	3.065347000	0.046174000
C	-9.703681000	1.005950000	-1.804980000	C	-9.671959000	1.072959000	-1.920377000
C	-10.675581000	3.142597000	-1.241404000	C	-10.800164000	3.120542000	-1.228152000
C	-10.180885000	4.223877000	0.887901000	C	-10.263953000	4.171304000	0.915635000
H	-9.517611000	0.194604000	-2.493219000	H	-9.453253000	0.301101000	-2.656266000
H	-11.905796000	4.295404000	-2.575948000	H	-12.181951000	4.241785000	-2.456131000
H	-10.963119000	2.113963000	-3.113822000	H	-11.077505000	2.149800000	-3.156064000
C	-10.911583000	5.316867000	0.499850000	C	-11.091967000	5.246032000	0.610751000
H	-9.683714000	4.227526000	1.845623000	H	-9.713867000	4.172033000	1.853869000
H	-10.986379000	6.171734000	1.158313000	H	-11.176282000	6.078564000	1.308989000
C	-11.557486000	5.341798000	-0.748713000	C	-11.808047000	5.263084000	-0.598190000
H	-12.136166000	6.208402000	-1.038376000	H	-12.465267000	6.100007000	-0.834867000
C	-11.433893000	4.277766000	-1.602089000	C	-11.653228000	4.222203000	-1.502110000
O	-6.560472000	1.449387000	0.718577000	O	-6.488759000	1.453527000	0.637723000
O	-8.307785000	-0.143863000	-0.242972000	O	-8.210354000	-0.119943000	-0.442451000
P	-6.703414000	0.101656000	-0.157217000	P	-6.617217000	0.149183000	-0.306040000
O	-6.136559000	-1.157477000	0.437854000	O	-6.029060000	-1.143753000	0.242011000
C	-6.055171000	0.578677000	-1.741691000	C	-5.922247000	0.698733000	-1.855851000
C	-5.117546000	1.246639000	-4.259018000	C	-4.943293000	1.503784000	-4.338954000
C	-5.449948000	-0.396400000	-2.535382000	C	-5.113677000	-0.172571000	-2.602138000
C	-6.198358000	1.887195000	-2.207840000	C	-6.247886000	1.973336000	-2.354625000
C	-5.726261000	2.213965000	-3.468525000	C	-5.754148000	2.367389000	-3.597538000
C	-4.978805000	-0.054492000	-3.792265000	C	-4.621605000	0.237173000	-3.839660000
H	-5.330582000	-1.405442000	-2.167237000	H	-4.861444000	-1.157219000	-2.212938000
H	-6.664129000	2.641386000	-1.591091000	H	-6.872130000	2.648435000	-1.774256000
H	-5.830114000	3.226704000	-3.832053000	H	-6.001961000	3.353794000	-3.986459000
H	-4.499271000	-0.804784000	-4.405091000	H	-3.986120000	-0.433802000	-4.415282000
H	-4.747079000	1.509401000	-5.240616000	H	-4.558232000	1.819351000	-5.307844000
Zn	-4.162343000	-1.602286000	0.737497000	Zn	-4.112024000	-1.497345000	0.742489000
Br	-3.562124000	-3.382794000	-0.735844000	Br	-3.179785000	-3.162026000	-0.733050000
Br	-2.951000000	0.442573000	0.92119900	Br	-2.816466000	0.422071000	1.372512000
**[$MnBr_2${O=PPh(BINOL)}$_2$] (sextet state, r^2SCAN-3c)**				**[$MnBr_2${O=PPh(BINOL)}$_2$] (octet state, r^2SCAN-3c)**			
H	-4.883010000	-0.169211000	5.391888000	H	-4.913989000	-0.238054000	5.412243000
C	-3.845300000	0.116110000	5.250956000	C	-3.880225000	0.057510000	5.262381000
H	-4.166302000	2.223568000	5.395915000	H	-4.213959000	2.159201000	5.508357000
C	-3.442488000	1.426370000	5.247461000	C	-3.485014000	1.375524000	5.321030000
C	-1.536792000	-0.640117000	4.897540000	C	-1.559126000	-0.680218000	4.884262000
C	-2.088387000	1.764181000	5.010969000	C	-2.130277000	1.728580000	5.107674000
C	-2.883061000	-0.885132000	5.063193000	C	-2.924836000	-0.928551000	5.031859000
C	-1.120875000	0.726600000	4.813947000	C	-1.157327000	0.708808000	4.864107000
C	-1.679143000	3.117640000	4.930616000	C	-1.733166000	3.087425000	5.083725000
H	0.941121000	0.323479000	4.286931000	H	0.888555000	0.350166000	4.264832000
C	-0.377532000	3.441736000	4.637225000	C	-0.437041000	3.437768000	4.789256000
H	-2.422722000	3.894394000	5.090225000	H	-2.482204000	3.849420000	5.283868000
H	-0.074241000	4.482480000	4.569831000	H	-0.144075000	4.483369000	4.766208000

C	0.566428000	2.420462000	4.399918000	C	0.506083000	2.436902000	4.485872000
H	1.587140000	2.683569000	4.137450000	H	1.519425000	2.716055000	4.211934000
C	0.206387000	1.096630000	4.486004000	C	0.152833000	1.106080000	4.518482000
C	-0.799908000	-2.734411000	3.798712000	C	-0.845373000	-2.827917000	3.767882000
C	-0.590069000	-1.769889000	4.763149000	C	-0.622818000	-1.775785000	4.757815000
C	1.194684000	-3.929298000	4.343749000	C	1.332096000	-3.764211000	4.154775000
C	0.534830000	-1.935525000	5.631246000	C	0.570883000	-1.885942000	5.567209000
C	0.074623000	-3.805638000	3.563125000	C	0.092221000	-3.753312000	3.453054000
C	1.443097000	-3.018758000	5.399031000	C	1.568205000	-2.860875000	5.225842000
C	0.764148000	-1.092175000	6.746698000	C	0.761675000	-1.115500000	6.716748000
H	-0.155173000	-4.507943000	2.768076000	H	-0.104008000	-4.464708000	2.657177000
H	3.252507000	-3.995441000	6.053424000	H	3.507005000	-3.646039000	5.712366000
H	1.892901000	-4.744667000	4.174907000	H	2.082471000	-4.508643000	3.909139000
C	1.855135000	-1.282651000	7.560956000	C	1.950455000	-1.204319000	7.479777000
H	0.062198000	-0.293390000	6.961909000	H	-0.015181000	-0.424498000	7.028854000
H	2.008794000	-0.628801000	8.414714000	H	2.071850000	-0.567492000	8.350544000
C	2.773332000	-2.322928000	7.304707000	C	2.936498000	-2.089989000	7.111372000
H	3.634494000	-2.455111000	7.953261000	H	3.858833000	-2.161779000	7.680071000
C	2.565375000	-3.176054000	6.248971000	C	2.740997000	-2.927263000	5.991390000
O	-3.340737000	-2.220322000	5.062693000	O	-3.375694000	-2.266794000	5.071383000
O	-1.933123000	-2.642605000	2.974718000	O	-2.004676000	-2.771784000	2.991562000
P	-3.379462000	-2.952440000	3.630959000	P	-3.455224000	-3.029037000	3.660413000
O	-4.416590000	-2.438907000	2.671312000	O	-4.482992000	-2.504285000	2.696536000
C	-3.524679000	-4.676323000	4.033104000	C	-3.636230000	-4.738254000	4.094844000
C	-3.642934000	-7.403426000	4.520535000	C	-3.795278000	-7.453835000	4.631268000
C	-4.190160000	-5.512444000	3.128847000	C	-4.384093000	-5.567179000	3.250169000
C	-2.913337000	-5.200548000	5.179438000	C	-2.960520000	-5.264322000	5.204341000
C	-2.978921000	-6.568002000	5.417122000	C	-3.047809000	-6.626231000	5.467001000
C	-4.247312000	-6.877885000	3.380459000	C	-4.462305000	-6.926479000	3.526948000
H	-4.658552000	-5.096502000	2.241469000	H	-4.898736000	-5.149527000	2.389760000
H	-2.397478000	-4.549139000	5.878496000	H	-2.381892000	-4.618667000	5.858315000
H	-2.511545000	-6.981804000	6.305415000	H	-2.531786000	-7.041653000	6.327201000
H	-4.766021000	-7.531393000	2.685824000	H	-5.045224000	-7.574384000	2.879654000
H	-3.691080000	-8.471451000	4.713116000	H	-3.859492000	-8.517389000	4.842936000
H	-5.541410000	1.445875000	2.935652000	H	-5.557535000	1.377465000	2.962269000
C	-6.617652000	1.588111000	2.971300000	C	-6.632129000	1.534299000	2.985984000
H	-6.775956000	1.649009000	5.101567000	H	-6.815489000	1.578598000	5.114605000
C	-7.304470000	1.695702000	4.153049000	C	-7.331675000	1.641356000	4.160287000
C	-8.696646000	1.866362000	1.692414000	C	-8.691264000	1.853214000	1.684477000
C	-8.713578000	1.835338000	4.160863000	C	-8.738652000	1.801098000	4.152265000
C	-7.334274000	1.668291000	1.769095000	C	-7.332905000	1.634765000	1.775944000
C	-9.428636000	1.900511000	2.921592000	C	-9.437523000	1.887383000	2.905038000
C	-9.434674000	1.881385000	5.379181000	C	-9.473893000	1.846778000	5.362134000
H	-11.402303000	1.952831000	2.031501000	H	-11.399365000	1.975498000	1.991811000
C	-10.805865000	1.956691000	5.382721000	C	-10.843826000	1.941467000	5.349812000
H	-8.878891000	1.841942000	6.312656000	H	-8.930178000	1.791189000	6.301868000
H	-11.349697000	1.985740000	6.322429000	H	-11.398619000	1.969940000	6.283111000
C	-11.513489000	1.975237000	4.162138000	C	-11.536141000	1.980779000	4.121007000
H	-12.599348000	2.004493000	4.170976000	H	-12.621510000	2.025223000	4.116964000
C	-10.844014000	1.948062000	2.961794000	C	-10.852491000	1.954801000	2.928673000
C	-9.165609000	1.006734000	-0.582856000	C	-9.144525000	1.020802000	-0.604113000
C	-9.349279000	1.988951000	0.369687000	C	-9.325974000	1.996887000	0.355127000
C	-10.609120000	2.062798000	-2.166707000	C	-10.552368000	2.111892000	-2.196161000
C	-10.141082000	3.124208000	0.005829000	C	-10.097346000	3.146370000	-0.007867000
C	-9.788008000	1.014731000	-1.840072000	C	-9.750457000	1.049277000	-1.869012000
C	-10.790859000	3.143407000	-1.270416000	C	-10.730555000	3.186463000	-1.291969000
C	-10.269100000	4.260163000	0.843245000	C	-10.220748000	4.276017000	0.838708000
H	-9.604637000	0.192412000	-2.524394000	H	-9.570077000	0.230669000	-2.558560000
H	-12.082183000	4.256666000	-2.593669000	H	-11.989385000	4.329728000	-2.620891000
H	-11.109368000	2.086525000	-3.131038000	H	-11.039856000	2.151612000	-3.166478000
C	-11.034782000	5.335284000	0.459609000	C	-10.966778000	5.365084000	0.455639000
H	-9.746510000	4.282226000	1.793768000	H	-9.710234000	4.282207000	1.796023000
H	-11.113885000	6.198997000	1.113626000	H	-11.042546000	6.223577000	1.116882000
C	-11.711937000	5.333143000	-0.778277000	C	-11.627902000	5.383955000	-0.790731000
H	-12.318890000	6.187545000	-1.063335000	H	-12.219450000	6.249260000	-1.075283000
C	-11.585202000	4.260938000	-1.626800000	C	-11.504859000	4.318136000	-1.647778000
O	-6.594466000	1.564296000	0.575630000	O	-6.580098000	1.529389000	0.590757000
O	-8.331217000	-0.086084000	-0.289584000	O	-8.330715000	-0.087069000	-0.310197000
P	-6.738872000	0.196418000	-0.257137000	P	-6.734815000	0.169855000	-0.254151000
O	-6.112585000	-1.026254000	0.353576000	O	-6.136634000	-1.066923000	0.356159000
C	-6.128583000	0.628103000	-1.870933000	C	-6.095039000	0.603309000	-1.856047000

C	-5.226798000	1.216533000	-4.427378000	C	-5.146313000	1.196086000	-4.394357000
C	-5.519887000	-0.372086000	-2.638488000	C	-5.490589000	-0.400675000	-2.621951000
C	-6.291053000	1.922732000	-2.380815000	C	-6.229966000	1.903996000	-2.358579000
C	-5.837119000	2.209011000	-3.662404000	C	-5.752581000	2.192400000	-3.631118000
C	-5.068048000	-0.070031000	-3.917256000	C	-5.015118000	-0.096467000	-3.891615000
H	-5.392241000	-1.373996000	-2.237466000	H	-5.384120000	-1.407264000	-2.226412000
H	-6.759099000	2.698088000	-1.782041000	H	-6.694781000	2.682100000	-1.760856000
H	-5.954995000	3.211570000	-4.062113000	H	-5.848885000	3.199517000	-4.025104000
H	-4.587241000	-0.840106000	-4.512679000	H	-4.537377000	-0.869592000	-4.485554000
H	-4.869573000	1.449160000	-5.426572000	H	-4.770654000	1.430406000	-5.386374000
Mn	-4.144839000	-1.515231000	0.822469000	Mn	-4.182855000	-1.588322000	0.847711000
Br	-3.321915000	-3.278341000	-0.724312000	Br	-3.387420000	-3.362809000	-0.700930000
Br	-2.898869000	0.623801000	1.038629000	Br	-2.903797000	0.529163000	1.084865000

$[MnBr_2\{O{=}PPh(BINOL)\}_2]_2$ (undecet state, r^2SCAN-3c)

H	-2.660861000	-0.646631000	-0.929510000
C	-2.824080000	0.243004000	-1.529937000
H	-2.011967000	-0.570407000	-3.323231000
C	-2.468295000	0.296417000	-2.852797000
C	-3.705313000	2.530928000	-1.629379000
C	-2.653027000	1.482259000	-3.602596000
C	-3.421954000	1.367305000	-0.944633000
C	-3.254636000	2.625110000	-2.984875000
C	-2.226388000	1.568102000	-4.950228000
H	-3.751246000	4.715069000	-3.262188000
C	-2.349460000	2.740910000	-5.653754000
H	-1.780534000	0.689313000	-5.408749000
H	-2.010690000	2.798566000	-6.684322000
C	-2.898030000	3.881317000	-5.030466000
H	-2.963734000	4.815559000	-5.581232000
C	-3.342090000	3.825866000	-3.730772000
C	-3.970659000	4.136835000	0.240689000
C	-4.433179000	3.629421000	-0.956779000
C	-5.693542000	5.788128000	0.378205000
C	-5.653402000	4.168911000	-1.476854000
C	-4.569723000	5.213651000	0.912825000
C	-6.271107000	5.274512000	-0.807956000
C	-6.306082000	3.617910000	-2.607626000
H	-4.128754000	5.566094000	1.839991000
H	-7.921493000	6.665310000	-0.818484000
H	-6.168474000	6.629350000	0.875812000
C	-7.478819000	4.159409000	-3.078018000
H	-5.878719000	2.750570000	-3.099471000
H	-7.966625000	3.716204000	-3.941532000
C	-8.060690000	5.278710000	-2.446194000
H	-8.983356000	5.699727000	-2.834965000
C	-7.470474000	5.818581000	-1.329916000
O	-3.789034000	1.261995000	0.411045000
O	-2.821255000	3.583842000	0.821358000
P	-2.947648000	2.113049000	1.487474000
O	-1.547916000	1.658066000	1.756676000
C	-4.023442000	2.167909000	2.903616000
C	-5.650237000	2.430392000	5.135163000
C	-3.444724000	2.302364000	4.171722000
C	-5.416046000	2.162254000	2.749710000
C	-6.223686000	2.293081000	3.872785000
C	-4.265758000	2.431034000	5.285236000
H	-2.363539000	2.311069000	4.287273000
H	-5.862078000	2.054487000	1.765733000
H	-7.304022000	2.284437000	3.762803000
H	-3.823951000	2.524454000	6.272623000
H	-6.287139000	2.529499000	6.009510000
Mn	-0.289850000	0.426183000	2.883043000
Br	1.863148000	-0.370422000	1.695563000
Br	0.733902000	2.414877000	4.195468000
Mn	0.337390000	-1.877939000	-0.186031000
Br	-1.512345000	-1.747682000	1.626262000
Br	-1.087492000	-3.231485000	-1.884554000
H	-4.066570000	-2.471074000	4.995877000
C	-4.461737000	-1.786083000	5.739226000
H	-6.486535000	-1.873932000	5.066836000
C	-5.789773000	-1.449416000	5.784583000

C	-3.987869000	-0.357179000	7.677786000
C	-6.268168000	-0.525822000	6.744716000
C	-3.590077000	-1.231111000	6.687675000
C	-5.359301000	0.052765000	7.687566000
C	-7.628889000	-0.132968000	6.761849000
H	-5.162826000	1.543557000	9.247946000
C	-8.076528000	0.816513000	7.647317000
H	-8.308789000	-0.588692000	6.046280000
H	-9.120848000	1.115065000	7.648776000
C	-7.171224000	1.425345000	8.542013000
H	-7.522135000	2.200592000	9.217250000
C	-5.846951000	1.055687000	8.561624000
C	-1.842595000	0.771420000	8.178667000
C	-2.995889000	0.168698000	8.641151000
C	-1.076336000	1.328720000	10.372474000
C	-3.157078000	0.049173000	10.058335000
C	-0.887333000	1.368103000	9.015491000
C	-2.191744000	0.654734000	10.925956000
C	-4.213175000	-0.694224000	10.641528000
H	-0.019284000	1.842427000	8.568372000
H	-1.620995000	1.018547000	12.975203000
H	-0.353828000	1.790638000	11.039893000
C	-4.332368000	-0.796459000	12.007183000
H	-4.928224000	-1.196651000	9.998375000
H	-5.144703000	-1.378303000	12.433368000
C	-3.406257000	-0.161016000	12.861269000
H	-3.518991000	-0.245255000	13.938242000
C	-2.354978000	0.544008000	12.328687000
O	-2.246146000	-1.636988000	6.632514000
O	-1.593837000	0.814114000	6.796700000
P	-1.150564000	-0.580957000	6.100918000
O	-1.145246000	-0.392758000	4.618217000
C	0.367473000	-1.183621000	6.812124000
C	2.769115000	-2.017805000	7.928479000
C	1.562449000	-0.996855000	6.108625000
C	0.372301000	-1.788669000	8.076309000
C	1.578110000	-2.203014000	8.628320000
C	2.761242000	-1.417898000	6.671425000
H	1.560096000	-0.524281000	5.130769000
H	-0.555057000	-1.940670000	8.620221000
H	1.586492000	-2.675362000	9.605913000
H	3.688599000	-1.278768000	6.124377000
H	3.708219000	-2.346735000	8.364441000
H	-0.334921000	-2.015787000	-4.769935000
C	0.588398000	-1.795038000	-5.296489000
H	0.579484000	-3.552683000	-6.508018000
C	1.102255000	-2.636099000	-6.248165000
C	2.456833000	-0.230851000	-5.602265000
C	2.331522000	-2.341691000	-6.883719000
C	1.278027000	-0.611943000	-4.992581000
C	3.039314000	-1.145628000	-6.540734000
C	2.890347000	-3.232608000	-7.831908000
H	4.877060000	-0.045953000	-6.860994000
C	4.114586000	-2.981916000	-8.399598000
H	2.334906000	-4.131460000	-8.087376000
H	4.539386000	-3.677837000	-9.117169000
C	4.834983000	-1.826983000	-8.030216000
H	5.816343000	-1.644049000	-8.458979000
C	4.310278000	-0.930485000	-7.129509000
C	3.314272000	1.413018000	-3.953466000
C	3.075973000	1.071185000	-5.270427000
C	4.356789000	3.487019000	-4.519028000
C	3.406649000	2.041043000	-6.271617000
C	3.954007000	2.596687000	-3.559488000
C	4.079403000	3.245259000	-5.885726000
C	3.042695000	1.883689000	-7.632420000
H	4.122319000	2.774961000	-2.502268000
H	4.946529000	5.098677000	-6.570777000
H	4.869302000	4.403051000	-4.237620000
C	3.374026000	2.835802000	-8.567027000
H	2.484949000	1.004032000	-7.935436000

H	3.077343000	2.697420000	-9.602852000
C	4.086706000	3.994871000	-8.193654000
H	4.349871000	4.733325000	-8.945423000
C	4.423355000	4.196420000	-6.877769000
O	0.662843000	0.249087000	-4.073607000
O	2.909202000	0.541938000	-2.928176000
P	1.315535000	0.516005000	-2.627317000
O	1.079106000	-0.529317000	-1.589018000
C	0.723578000	2.142079000	-2.210951000
C	-0.089907000	4.691106000	-1.479282000
C	0.554873000	2.454479000	-0.857932000
C	0.486924000	3.103544000	-3.201889000
C	0.077263000	4.376666000	-2.827155000
C	0.149294000	3.733464000	-0.496968000
H	0.736320000	1.701918000	-0.095795000
H	0.608131000	2.857084000	-4.251853000
H	-0.119644000	5.122514000	-3.591007000
H	0.009982000	3.973710000	0.551957000
H	-0.414624000	5.687968000	-1.193756000
H	5.113846000	-2.141502000	0.993251000
C	5.343498000	-1.864521000	-0.030552000
H	6.315656000	-0.025346000	0.451822000
C	6.000548000	-0.701600000	-0.338254000
C	5.204942000	-2.471762000	-2.406808000
C	6.258778000	-0.354876000	-1.685626000
C	4.960650000	-2.722798000	-1.071140000
C	5.836470000	-1.229905000	-2.737669000
C	6.915306000	0.856828000	-2.013665000
H	5.665604000	-1.434199000	-4.884501000
C	7.125823000	1.211323000	-3.323187000
H	7.239771000	1.505881000	-1.204000000
H	7.625093000	2.145341000	-3.564400000
C	6.662569000	0.375979000	-4.360158000
H	6.792788000	0.682691000	-5.394674000
C	6.029908000	-0.810347000	-4.075941000
C	3.472615000	-3.904610000	-3.442421000
C	4.777756000	-3.452292000	-3.431145000
C	3.805539000	-5.304368000	-5.347100000
C	5.676382000	-4.015728000	-4.394067000
C	2.967321000	-4.815883000	-4.380608000
C	5.169429000	-4.929733000	-5.373324000
C	7.067812000	-3.745613000	-4.386833000
H	1.923419000	-5.106656000	-4.320178000
H	5.645896000	-6.141469000	-7.093194000
H	3.434938000	-5.999494000	-6.095273000
C	7.898911000	-4.296399000	-5.333177000
H	7.479940000	-3.100176000	-3.618519000
H	8.962734000	-4.078151000	-5.305808000
C	7.386371000	-5.149092000	-6.333906000
H	8.054141000	-5.567557000	-7.081422000
C	6.050462000	-5.465525000	-6.343927000
O	4.328367000	-3.924101000	-0.698674000
O	2.575774000	-3.437196000	-2.467664000
P	2.748703000	-4.060455000	-0.984282000
O	1.863009000	-3.306701000	-0.040166000
C	2.479037000	-5.819552000	-1.000558000
C	1.941382000	-8.538497000	-1.118101000
C	1.220650000	-6.294139000	-0.612421000
C	3.469766000	-6.701946000	-1.449953000
C	3.193323000	-8.062639000	-1.504229000
C	0.957939000	-7.657477000	-0.673387000
H	0.458125000	-5.599994000	-0.271069000
H	4.445512000	-6.331239000	-1.748871000
H	3.958044000	-8.753041000	-1.846877000
H	-0.015162000	-8.031604000	-0.370221000
H	1.732311000	-9.603684000	-1.161794000
